# Spatial Transcriptomics of Immune Cell Distribution in Non-Small Cell Lung Cancer Identifies Tertiary Lymphoid Structures and Its Density and Area Fraction Were Associated with Neoadjuvant Therapy Response

**DOI:** 10.3390/cancers18132141

**Published:** 2026-07-02

**Authors:** Zelin Jin, Ziqiang Chen, Dongxian Jiang, Yingyong Hou, Yun Liu

**Affiliations:** 1 MOE Key Laboratory of Metabolism and Molecular Medicine, Department of Biochemistry and Molecular Biology, School of Basic Medical Sciences, Fudan University, Shanghai 200032, China; 17111010003@fudan.edu.cn; 2 MOE Key Laboratory of Metabolism and Molecular Medicine, Department of Biochemistry and Molecular Biology, Shanghai Xuhui Central Hospital, Fudan University, Shanghai 200031, China; ziqiangchen21@m.fudan.edu.cn; 3 State Key Laboratory of Medical Neurobiology and MOE Frontiers Center for Brain Science, Institutes of Brain Science, Fudan University, Shanghai 200032, China; 4 Department of Pathology, Zhongshan Hospital, Fudan University, Shanghai 200032, China; jiang.dongxian@zs-hospital.sh.cn

**Keywords:** spatial transcriptomics, non-small cell lung cancer (NSCLC), tertiary lymphoid structure (TLS), neoadjuvant treatment

## Abstract

Background: Non-small cell lung cancer (NSCLC) remains one of the leading causes of cancer-related mortality worldwide over the past decade. Single-cell sequencing loses spatial location information and potential cell–cell interactions, making it difficult to interpret molecular features or biological phenomena. Tertiary lymphoid structures (TLSs) inherently require such spatial immune cell distribution information. Although associations between TLS and response to immune checkpoint inhibitors (ICIs) or chemotherapy have been reported, the relationship between TLS and neoadjuvant therapy (ICI combined with chemotherapy) remains unclear. Methods: We performed spatial transcriptomics on NSCLC samples (including one lung squamous cell carcinoma (LUSC) and one lung adenocarcinoma (LUAD)). Multiplex immunohistochemistry (mIHC) was used to identify the TLS, while immunohistochemistry staining (IHC) was used to identify the TLS status and cell characteristics. We evaluated the associations between (mature) TLS density, area proportion and patients’ responses in 66 patients. Results: Heterogeneity of immune cells in NSCLC was found. Gene ontology analysis and cell score comparison identified TLS with activated B and T cells inside, while plasma cells and macrophages were mainly distributed outside TLS. Four genes from antigen-presenting machinery (TAP1, TAP2, B2M, TAPBP) were more highly expressed inside TLS than outside them. Also, TLS exhibited heterogeneity, with both mature and immature TLS. Mature TLS showed an average area of 62,387.43 μm^2^, while the immature TLS showed 51,189.90 μm^2^. The Spearman correlation coefficient of B-cell number and mTLS area showed r = 0.900. TLS density and mature TLS (mTLS) density in the tumor bed were 1.95 ± 0.95 TLS/10 mm^2^ (mean ± SD, *n* = 34) and 1.13 ± 0.77 mTLS/10 mm^2^, significantly higher than that in the non-responder group (1.18 ± 1.15 TLS/10 mm^2^, 0.70 ± 0.90 mTLS/10 mm^2^, mean ± SD, *n* = 32) separately. B cells belonging to TLS had a significantly higher density (71.32 ± 55.71 cells/mm^2^, mean ± SD, *n* = 34) in the responder group than the non-responder group (61.33 ± 111.95 cells/mm^2^, mean ± SD, *n* = 32) normalized to the tumor bed area. Conclusions: Spatial transcriptomics reveals immune cell heterogeneity and distribution patterns in the NSCLC tumor bed, with activated B and T cells localized inside and plasma cells/macrophages outside. Antigen-presenting machinery (APM)-related genes were highly expressed in TLS accompanied by a high expression of upstream and downstream genes of MHC class I. mTLS have a larger area by mainly containing more B cells. The responder group had a significantly higher (mature) TLS density and larger (mature) TLS area proportion compared with the non-responder group, suggesting their potential function in anti-tumor effect in neoadjuvant treatment.

## Simple Summary

Non-small cell lung cancer (NSCLC) remains a challenge to treat, and not all patients benefit from neoadjuvant therapy. This study examined whether the distribution of immune cells and the presence of tertiary lymphoid structures (TLSs), clusters of immune cells within tumors, are linked to treatment outcomes. Using spatial transcriptomics, we found that activated B and T cells were located inside TLS, whereas macrophages and plasma cells were predominantly outside. With multiplex staining, we compared samples from responders and non-responders. Responders had higher TLS density, especially mature ones, within the tumor bed, and these TLS contained more B cells. Genes involved in antigen presentation were highly active inside TLS. Our results suggest that TLS density, area proportion in the tumor bed, and TLS maturity are associated with favorable treatment response, and they offer new clues about how TLS contribute to anti-tumor immunity.

## 1. Introduction

Non-small cell lung cancer (NSCLC), mainly comprising lung squamous cell carcinoma (LUSC) and lung adenocarcinoma (LUAD), accounts for about 85% of all lung cancers [1]. Over the past 10 years, systematic early screening and novel surgical/radiotherapeutic techniques have improved NSCLC prognosis, yet NSCLC remains the leading cause of cancer-related deaths worldwide [2]. Future therapeutic strategies will require better predictive clinical/genetic markers and a deeper biological understanding of NSCLC.

Single-cell sequencing has profiled NSCLC cell composition and states but lacks spatial localization data [3,4]. Interactions among cells can drive the same cell type into distinct functional states, ultimately giving rise to divergent immune microenvironments [5]. One notable example of these microenvironmental differences is tertiary lymphoid structures (TLSs), which were first identified in NSCLC [6]. TLS are defined as aggregates mainly of T and B lymphocytes arising in non-lymphoid tissues, observed in chronically inflamed tissues, rejected transplanted organs, autoimmune lesions, and the tumor microenvironment [7].

In many tumors (such as melanoma [8], renal cell carcinoma [9], sarcoma [10], urothelial carcinoma [11]), TLS have been proven to be associated with the therapeutic efficacy of immune checkpoint therapy, where they are thought to serve as local hubs for the initiation and regulation of anti-tumor immune responses. However, at the same time, it has been reported that chemotherapy inhibits germinal center formation in TLS and leads to a poorer clinical outcome in LUSC patients [12].

Also, studies in hepatocellular carcinoma have revealed that TLS at different anatomical locations are associated with patient prognosis [13], suggesting that the spatial distribution of TLS represents critical information linked to therapeutic efficacy.

In this study, using spatial transcriptomic sequencing, we found that in NSCLC patients who respond to neoadjuvant therapy, the tumor bed exhibits heterogeneous immune cell distribution and contains TLS, with activated B and T cells localized inside and plasma cells/macrophages outside. Our findings show that genes related to the antigen-presenting machinery (APM) were upregulated in TLS compared to other regions, along with elevated upstream and downstream genes of MHC class I, suggesting a mechanism for cell activation within TLS. TLS and mature TLS density and area proportion, particularly B-cell enrichment within mature TLS, are associated with favorable neoadjuvant therapy response in NSCLC.

## 2. Materials and Methods

### 2.1. Pathological Samples

Spatial transcriptomic sequencing was performed on tumor samples obtained from Zhongshan Hospital Affiliated to Fudan University. The tumor tissues from the two patients were both surgical samples obtained after neoadjuvant treatment with PD-1 antibody combined with chemotherapy drugs. Both cases had ≤10% residual tumor of the tumor bed and were evaluated by an experienced pathologist; the patients were classified as responders [14].

The first case was lung squamous cell carcinoma (LUSC), and the second was lung adenocarcinoma (LUAD). All spatial transcriptomic analyses were confined to the tumor bed regions of the tissue sections.

In H&E-stained pathological sections, those with residual tumor area no more than 10% of the tumor bed area as determined by pathologists are defined as the neoadjuvant therapy responder group, while the rest are classified as the non-responder group.

There were 66 samples in this analysis cohort, containing two spatial transcriptome sequencing cases. Among them, the responder group included 34 individuals (32 males and 2 females) while the non-responder group included 32 individuals (28 males and 4 females). The average age of the responder group was 63.5 ± 7.9 and that of the responder group was 63.5 ± 7.6. Detailed patient characteristics are summarized in Table S1, Summary Table of Patient Characteristics. All samples were obtained after neoadjuvant treatment with PD-1 antibody combined with chemotherapy drugs.

Informed consent was obtained from all patients involved in the study. The study was approved by the Medical Ethics Committee of Zhongshan Hospital, Fudan University (approval number: B2022-632, January 2023).

### 2.2. Spatial Transcriptome Sequencing

In brief, a 6.5 mm × 6.5 mm region was captured by using the Visium CytAssist Spatial Gene Expression for FFPE, Human Transcriptome kit from 10× Genomics (Pleasanton, CA, USA), followed by library construction. After library preparation, paired-end 150 bp (2 × 150 bp) sequencing was performed on the NovaSeq platform from Illumina (San Diego, CA, USA). All library construction and sequencing procedures were carried out by Shanghai Neo-Bio Co., Ltd. (Shanghai, China).

### 2.3. Quality Assessment of Spatial Transcriptome Sequencing

Quality control of FFPE samples from a LUSC patient showed DV200 > 30% and Q30 Bases in Probe Read of 96.5%, both within acceptable limits. A total of 4738 spatial transcriptome spots (each representing 1–10 cells) were detected, averaging 3700 genes with 72,925.4 reads per spot (Figure S1A). As expected, mitochondrial and red blood cell gene proportions were very low (Figure S1B,D), and ribosomal mRNA was undetectable (Figure S1C). After filtering out spots with nFeature ≤ 200 or percent mt > 10% per spot [15], 4729 spots remained for further analysis.

The LUAD sample also showed DV200 > 30% and Q30 Bases in Probe Read of 96.5%, both within acceptable limits. A total of 4914 spatial transcriptome spots were detected, averaging 5898 genes with 74,748 reads per spot (Figure S2A). As expected, mitochondrial and red blood cell gene proportions were very low (Figure S2B,D), and ribosomal mRNA was undetectable (Figure S2C). After filtering out spots with nFeature ≤ 200 or percent mt > 10% per spot, 4913 spots remained for further analysis.

### 2.4. Bioinformatics Analysis

Data analysis was based on R language (version 4.2.2). SpaceRanger 2.0.1 was used to transform the data. Seurat package was used to analyze and visualize the spatial transcriptomic data. Data was normalized by SCTransform (version 0.4.3). Cell scores were calculated using the Addmodulescore function and projected onto the H&E-stained images by SpatialDimplot. The functions AddModuleScore and SpatialDimPlot are from the Seurat package (version 4.3.0).Dimensionality reduction and visualization were performed using Loupe Browser 6 (10× Genomics). The t-SNE algorithm was applied for a two-dimensional projection of the data. K-means clustering was used to partition cells into 8 clusters, in LUSC and LUAD. The resulting cluster labels were overlaid on the t-SNE plot for display.

### 2.5. Hematoxylin-Eosin Staining (H&E)

Staining was performed on 4-µm section slides of FFPE tumor tissues. Sections were baked at 65 °C for 2 h, deparaffinized in xylene (3 × 10 min), and rehydrated through graded ethanol. Hematoxylin staining (5 min) was followed by differentiation in 0.1% HCl-ethanol (10 s), bluing in PBS (1 min), and eosin staining (2 min). After two quick dips in 70% ethanol (each < 1 min), sections were dehydrated, cleared in xylene, and mounted with neutral balsam.

### 2.6. Multiplex Immunohistochemistry (mIHC)

Multiplex immunohistochemistry staining was performed on section slides of FFPE tumor tissues of all 66 patients within the cohort, including the two samples used in spatial transcriptome sequencing. Mouse or rabbit anti-human monoclonal antibodies were used for staining as follows: anti-HuCD20 (Invitrogen, Carlsbad, CA, USA), anti-CD3 (Abcam, Cambridge, UK), and anti-HLA-A (Abclonal, Woburn, MA, USA). For plasma cell staining, anti-CD138 (Abcam) was used. Three wavelengths of Opal 520/570/690 (AKOYA) were used for staining. The stained slides were blocked with fluorescence mounting medium (with DAPI) and visualized with the Vectra Polaris image system (Perkin Elmer, Shelton, CT, USA). Based on the results of H&E staining, experienced pathologists delineated the tumor bed area.

TLS areas were manually segmented based on CD20 and CD3 staining with QuPath-0.3.2. We only included TLS with total cells ≥ 120, B cells ≥ 20, area ≥ 10,000 μm^2^, which are more stringent than those reported in the literature [16,17]. This stringent threshold minimizes false-positive TLS identification and reduces subjective bias in evaluation.

### 2.7. Immunohistochemistry Staining (IHC)

Immunohistochemistry staining of CD23 was performed on section slides of FFPE tumor tissues of 64 patients from the cohort. CD68 staining was performed on 1 LUSC and 1 LUAD that responded to the neoadjuvant therapy selected randomly. Antibodies were used as follows: CD68 (PTM Bio) (Hangzhou, China) and CD23 (Shanghai Long Island Antibody Diagnostica Inc.) (Shanghai, China). Mature TLS were defined by the presence of at least one CD23^+^ cell exhibiting the FDC morphology manually [16].

### 2.8. Statistical Analysis

Hypothesis tests used to calculate *p* values were specified at corresponding figure legends. *p* < 0.05 was considered significant. The TLS, area, number of T and B cells, and total cell count were all obtained by QuPath-0.3.2. segmentation. A total of 2339 TLS were included. The data were recorded and organized using an Excel spreadsheet, and the graphs were created using GraphPad Prism 8.0.2.

## 3. Results

### 3.1. Spatial Transcriptomic Analyses Revealed That the LUSC Patient Who Received Effective Neoadjuvant Therapy Exhibited Heterogeneity of Immune Cells in the Tumor Bed

**Figure 1:**
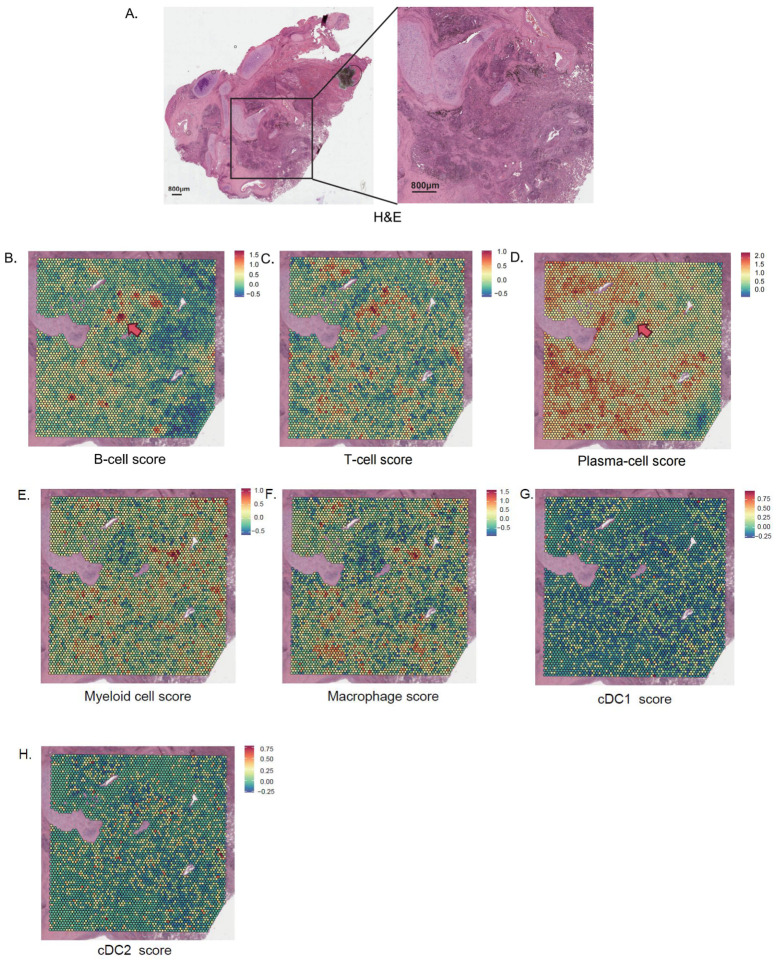
Spatial distribution of different immune cell scores overlaid on the H&E-stained tissue section for a single LUSC patient responding to neoadjuvant therapy. (**A**) Hematoxylin and eosin (H&E) staining of LUSC tumor bed showing the location of spatial transcriptomics sequencing. Scale bars: 800 μm for both the main image and inset. (**B**) Spatial distribution of B-cell scores overlaid on the H&E-stained tissue section. The red arrow shows the area with high B-cell scores, (**C**) T-cell scores, (**D**) plasma cell scores, (**E**) myeloid cell scores, (**F**) macrophage scores, (**G**) conventional dendritic cell type 1 (cDC1) scores, and (**H**) cDC2 scores.

Since each spot might contain from 1 to 10 cells, directly naming the cell types for each spot was not reasonable. To investigate the distribution of immune cells in the detected region, we scored the gene expression patterns of a series of immune cells and projected them onto the H&E-stained images (Figure 1A). For B cells, *MS4A1* (CD20), *CD79A*, *CD79B*, *IGHM*, and *IGHD* were used [18] (Figure 1B). For T cells, *CD3D*, *CD3E*, *CD3G*, *CD2*, and *TRBC2* [18] were used (Figure 1C). The distribution of B and T cells in the tumor bed showed a distinct clustering pattern.

*SLAMF7*, *DERL3*, *JSRP1*, and *IGHG1* [19] were used to identify the location of plasma cells, which were developed from B cells. Interestingly, the distribution of plasma cells is different from B cells (Figure 1D, red arrow). Myeloid cells, which are located by *LYZ*, *CD14*, *FCGR3A*, *CD163*, and *CSF1R* [18] also showed a distinct clustering pattern (Figure 1E). Further subdividing the myeloid cells into macrophages (Figure 1F) and two types of dendritic cells (Figure 1G,H) shows that only the macrophage has a distinct clustering.

For CD4^+^ and CD8^+^ T cells, as well as M1 and M2 macrophages, the spatial transcriptomic data resolution is insufficient to distinguish them spatially. Therefore, no further discussion is provided.

### 3.2. LUSC Patient Who Responded to Neoadjuvant Therapy Has TLS in the Tumor Bed and Can Be Located by Spatial Transcriptome Sequencing

**Figure 2:**
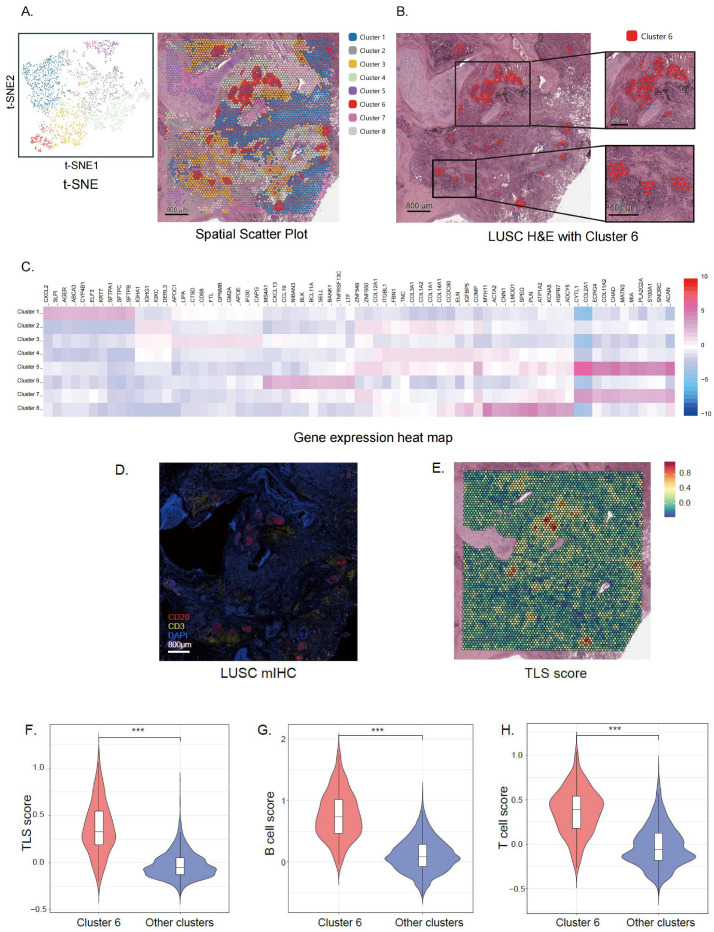
Spatial transcriptomics identifies Cluster 6 within a tertiary lymphoid structure (TLS) area in the LUSC patient who responded to neoadjuvant therapy. (**A**) K-means clustering was used to divide cells into eight clusters, and the resulting cluster labels were overlaid on the t-SNE plot (**A, left**) and H&E-stained section (**A, right**) for display. (**B**) Cluster 6 overlaid on the H&E-stained section. (**C**) Heatmap of differentially expressed genes for eight clusters in LUSC. Rows represent clusters and columns represent individual genes. The color scale indicates scaled expression, from low (blue) to high (red). (**D**) Multiplex immunohistochemistry (mIHC) staining on a serial section of LUSC. CD20 (B cells) in red, CD3 (T cells) in yellow, and nuclei were stained with DAPI (blue). Scale bar: 800 μm. (**E**) TLS score overlaid on the H&E-stained section. (**F**–**H**) Score comparison between Cluster 6 and other clusters. (**F**) TLS score comparison, (**G**) B-cell score comparison, (**H**) T-cell score comparison. In the boxplot, the center line denotes the median, and the box bounds represent the interquartile range. Wilcoxon rank-sum test was used; *** *p* <0.001.

To get a more detailed picture of the cell distribution within the tumor bed, the t-SNE algorithm was applied for a two-dimensional projection of the data. K-means clustering was used to divide cells into eight clusters, and the resulting cluster labels were overlaid on the t-SNE plot (Figure 2A, left) and spatial cluster plot (Figure 2A right) for display. Instead of representing the entire cluster by a single cell type, we rely on a combination of morphological features (Figure 2B), gene expression heatmaps (Figure 2C), and the SingleR reference-based method to annotate distinct major cell clusters.

Clusters 1, 2 and 3 are mainly composed of macrophages and tissue stem cells. Also, immunoglobulin specifically expressed by plasma cells was highly expressed in Cluster 3, implying the presence of plasma cells (Figure 2C). Cluster 4 is mainly composed of smooth muscle cells and tissue stem cells. Clusters 5 and 7 are mainly composed of chondrocytes. Cluster 6 is mainly composed of B cells and T cells. Chemokines (such as CXCL13) and selectins (such as SELL) were detected inside the TLS, which exhibit the characteristics of TLS. Cluster 8 is mainly composed of tissue stem cells.

A series of verifications were carried out to prove that Cluster 6 is contained within the TLS. First, multiplex immunohistochemistry was performed to detect CD20^+^ B cells and CD3^+^ T cells, a combination that serves as the gold standard for identifying TLS [20] (Figure 2D). The staining results revealed that the distribution of Cluster 6 was nested within the B- and T-cell aggregation regions, and their positions were highly consistent. The TLS signature score is a validated method for accurately localizing TLS in the spatial transcriptome sequencing samples [8]. Based on the expression levels of *CCL19*, *CCL21*, *CXCL13*, *CCR7*, *CXCR5*, *SELL*, and *LAMP3*, we further calculated a global TLS score and mapped it onto the H&E-stained section (Figure 2E). The distribution heatmap of TLS scores across the samples showed that Cluster 6 was close to regions with high scores.

Further quantification of the above results revealed that the TLS score in Cluster 6 was significantly higher than that in other regions (Figure 2F). B- and T-cell scores were also significantly higher than those in other regions, which is consistent with the definition of TLS (Figure 2G,H).

Taken together, our findings indicate that Cluster 6 is spatially located within the TLS and is transcriptionally consistent with it.

### 3.3. Activated B and T Cells Localize Inside the TLS, While Plasma Cells and Macrophages Reside Outside

**Figure 3:**
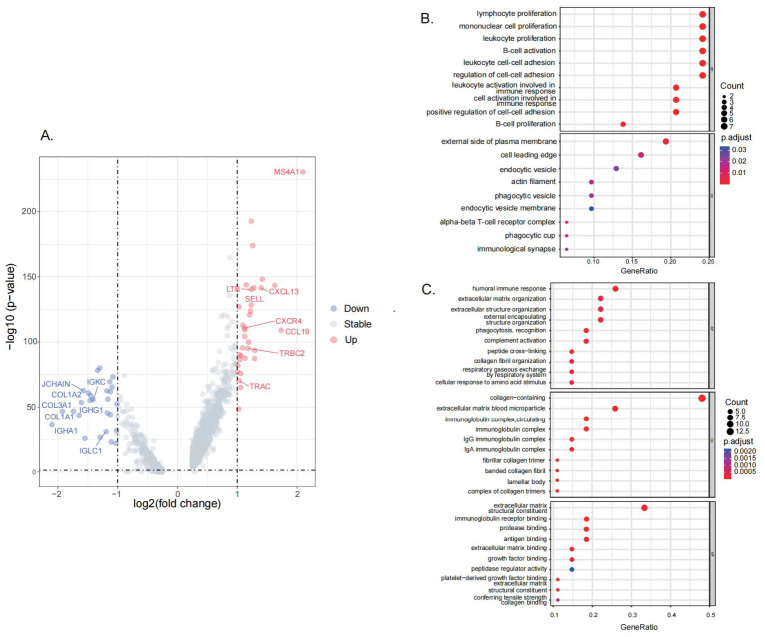

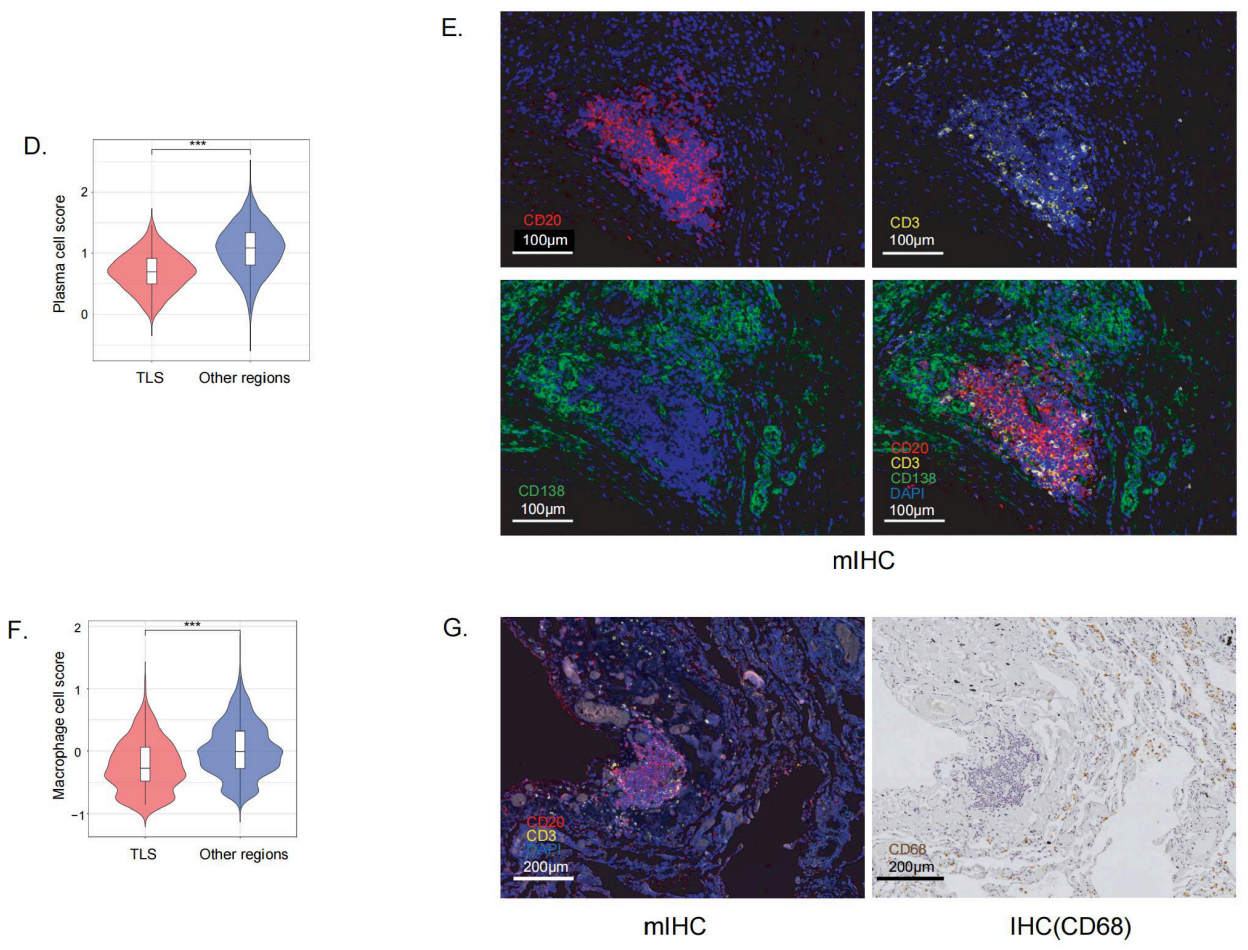
Activated B and T cells localized inside TLS while plasma cells and macrophages reside outside in the LUSC patient who responded to neoadjuvant therapy. (**A**) Volcano plot of differentially expressed genes between TLS and other regions. Red dots denote genes significantly upregulated in TLS (|log_2_(fold change)| ≥ 1, adjusted *p* < 0.05); blue dots denote genes significantly downregulated in TLS; gray dots denote non-significance. |log_2_FC| = 1 (vertical dashed line) and *p* = 0.05 (horizontal dashed line, on the −log_10_ scale). (**B**,**C**) Gene ontology (GO) enrichment analysis of genes (**B**) upregulated in TLS and (**C**) downregulated in TLS (adjusted *p* < 0.05). (**D**) Violin plot for plasma cell score comparison between TLS and other regions. In the box, the center line denotes the median, and the box bounds represent the interquartile range. Wilcoxon rank-sum test was used; ***: *p* < 0.001. (**E**) mIHC staining on an individual LUSC specimen responded to neoadjuvant therapy. CD20 (B cells, top left) in red, CD3 (T cells, top right) in yellow, CD138 (plasma cells, button left) in green and nuclei were stained with DAPI (blue). Scale bar: 100 μm. (**F**) Violin plot for macrophage score comparison between TLS and other regions. In the box, the center line denotes the median, and the box bounds represent the interquartile range. Wilcoxon rank-sum test was used; ***: *p* < 0.001. (**G**) mIHC staining (left) and CD68 immunohistochemistry staining (brown, right) on an individual LUSC specimen responded to neoadjuvant therapy. CD20 in red, CD3 in yellow and nuclei were stained with DAPI (blue). Scale bar: 200 μm.

To investigate the differences between the TLS and outside regions in the tumor bed of LUSC patient, we conducted an analysis of differentially expressed genes inside and outside Cluster 6, which will be marked as TLS and non-TLS area. The volcano plot shows a total of 32 genes upregulated and 27 genes downregulated inside TLS (Figure 3A). Since the TLS is a tissue structure composed of B and T lymphocytes, naturally, we found that the specific expression genes of these two types of cells were highly expressed within TLS. For example, B-cell-specific genes (e.g., MS4A1) and T-cell-specific genes, including the T-cell receptor subunits TRAC and TRBC2, were highly expressed within this region. Besides chemokines (like *CXCL13*) or selectins (like *SELL*) which are specific to TLS, we also found *LTB* was highly expressed inside TLS. *LTB* is a member of the TNF superfamily, localizes to the cell surface and forms a heterotrimer with *LTA* [21]. It serves as a canonical marker for lymphoid tissue inducer (LTi) cells during TLS neogenesis and is additionally expressed on B cells. Such gene shows that immune cells in these TLS were in an activated situation.

Genes that were downregulated within TLS were highly expressed outside TLS; these included collagen-related genes and secretory immunoglobulin-related genes (Figure 3A). *COL1A1* and *COL1A2* form the type I collagen heterotrimer, and *COL3A1* forms the type III collagen homologous trimer. Type I collagen, together with type III collagen, provide a structural framework in the bronchi, interstitium, and alveolar wall, serving as a primary contributor to pulmonary mechanics and conferring mechanical stability [22]. The histological cell distribution might be responsible for our result. *COL1* is a specific biomarker for fibroblasts in which plasma cells can spread along in renal cell cancer (RCC) [23]. The high expression of secretory immunoglobulin outside the TLS indicates that after B cells develop into plasma cells, they do not exist within the TLS but migrate outside it. The high expression of *COL1* and secretory immunoglobulin-related genes at the same region may potentially support the conclusion in LUSC.

Then, gene ontology (GO) enrichment analysis was conducted on the genes highly expressed inside TLS versus outside it to identify the biological clusters associated with each gene set (Figure 3B). The analysis revealed that genes upregulated within TLS were predominantly enriched in immune cell activation and proliferation, such as “B cell activation” and “B cell proliferation” enriched in Biological Processes (BPs). “Alpha-beta T cell receptor complex” enriched in cellular component (CC) suggested that T cells undergo physiological activation rather than being in a resting state.

Also, enrichments of “positive regulation of cell-cell adhesion” in BP (Figure 3B top) and “Immunological synapse” in CC (Figure 3B top) were observed. These enrichments may serve as the basis of the activation and proliferation of cells within the TLS.

Among genes highly expressed outside TLS, the GO enrichment analysis revealed BPs including a humoral immune response and extracellular matrix organization, and CC including immunoglobulin complex (especially IgG and IgA). During the development of B cells, they will successively express IGHM, IGD, IGHG (1–4), IGHE or IGHA [24]. The results showed that the highly expressed immunoglobulins outside TLS were all at an advanced stage of development.

After discovering these differences, we compared the score distribution of plasma cells inside versus outside TLS (Figure 3D). The plasma cell score was significantly lower within TLS than that outside TLS. To support this result, an additional specimen from a different LUSC patient who responded to neoadjuvant therapy was used for mIHC staining (Figure 3E). CD138^+^ [25,26] cells (marked green) were predominantly localized to outside the CD20^+^ B cells (marked red) and CD3^+^ T cells (marked yellow), consistent with the spatial transcriptome analysis (Figure 3E).

Our previous results in Figure 1F show that the distribution of macrophages exhibited a certain pattern. Here, we further compared the location of macrophages with TLS. It was found that the macrophages score outside the TLS was significantly higher than that within the TLS (Figure 3F). Additional specimen IHC staining with CD68 [25] supports this conclusion independently (Figure 3G).

In conclusion, activated B and T cells localize inside TLS in the tumor bed of an effectively treated LUSC, while plasma cells and macrophages reside outside.

### 3.4. Similar Findings in LUAD: Tumor Bed Shows Heterogeneous Immune Cell Distribution and Presence of TLS, While Plasma Cells and Macrophages Are Predominantly Localized Outside TLS

To validate the generalizability of the above findings, we performed spatial transcriptome sequencing on a tumor sample obtained from a LUAD patient who had received neoadjuvant therapy and responded to treatment. For the quality assessment of the LUAD sample, see Figure S2.

The distribution of B cells (Figure S3A), T cells (Figure S3B), plasma cells (Figure S3C) and myeloid cells (Figure S3D), especially macrophages (Figure S3E) in LUAD tumor bed show a distinct distribution pattern. Figure S3, at the same time, DC cells (Figure S3F,G) do not show any distinct spatial distribution characteristics.

**Figure 4:**
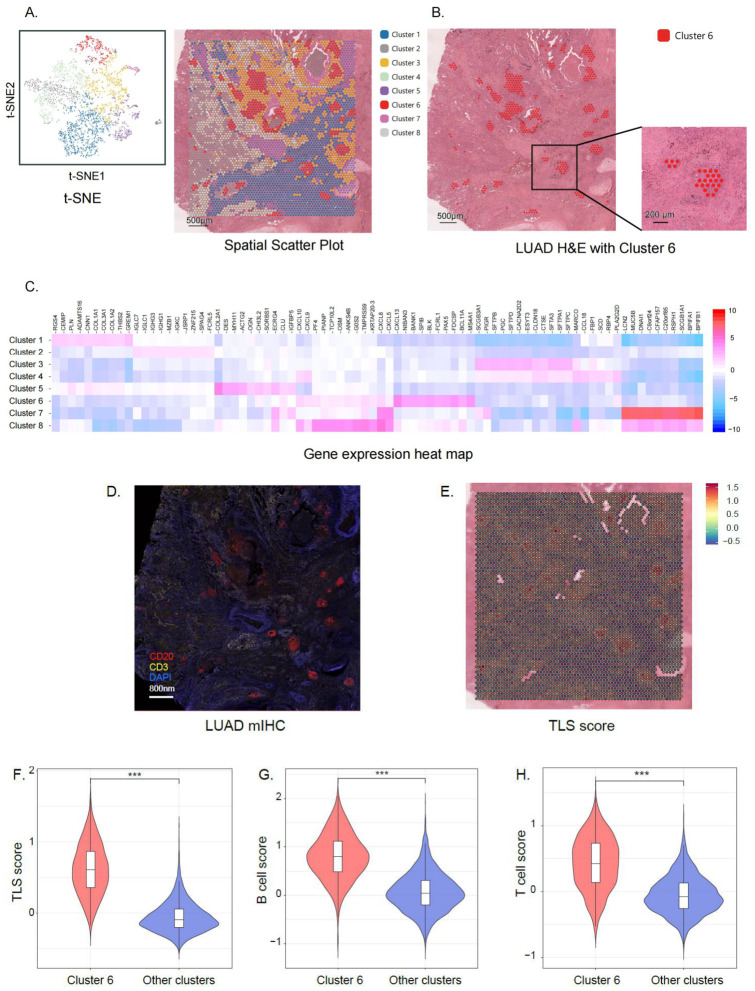
Spatial transcriptomics identifies a cluster (cluster 6) within a TLS in a LUAD patient who responded to neoadjuvant therapy, analogous to the observation in the LUSC patient (Figure 2). (**A**) K-means clustering was used to divide cells into eight clusters, and the resulting cluster labels were overlaid on the t-SNE plot (**A**, **left**) and H&E-stained section (**A**, **right**) for display. (**B**) Cluster 6 overlaid on the H&E section. (**C**) Heatmap of differentially expressed genes for eight clusters in LUAD. Rows represent clusters and columns represent individual genes. The color scale indicates scaled expression, from low (blue) to high (red). (**D**) Multiplex immunohistochemistry (mIHC) staining on serial section of LUAD. CD20 (B cells) in red, CD3 (T cells) in yellow, and nuclei were counterstained with DAPI (blue). Scale bar: 800 μm. (**E**) TLS score overlaid on the H&E section. (**F**–**H**) Violin plot for score comparison between Cluster 6 and other clusters. (**F**) TLS score comparison, (**G**) B-cell score comparison, (**H**) T-cell score comparison. In the boxplot, the center line denotes the median, and the box bounds represent the interquartile range. Wilcoxon rank-sum test was used; ***: *p* < 0.001.

The same analytical approach was applied to the LUAD specimen for a two-dimensional projection using t-SNE (Figure 4A left), coupled with k-means clustering to divide cells into eight clusters and the spatial cluster plot for display (Figure 4A right). We rely on a combination of morphological features (Figure 4B), gene expression heatmaps (Figure 4C), and the SingleR reference-based method to annotate distinct major cell clusters as before.

Cluster 1 is mainly composed of fibroblasts and macrophages, while Cluster 2 is mainly composed of plasma cells and macrophages. Cluster 3 is mainly composed of tissue stem cells and macrophages. Clusters 4 and 8 are mainly composed of macrophages. Cluster 5 is mainly composed of chondrocytes. Cluster 6 is mainly composed of B cells and T cells, and expresses a set of chemokines and selectins. Cluster 6 also exhibits the characteristics of TLS coincidentally. Cluster 7 is mainly composed of epithelial cells.

To validate that Cluster 6 was contained within TLS, mIHC (Figure 4D) and global TLS score mapping on the H&E-stained section were used (Figure 4E). Similar to the LUSC specimen, the distribution of Cluster 6 was nested within the B- and T-cell aggregation regions, and their positions were highly consistent with high TLS score regions (Figure 4B,D,E).

Further quantification of the above results revealed that the TLS score in Cluster 6 was significantly higher than that in other regions (Figure 4F). B- and T-cell scores were also significantly higher than that in other regions, which is consistent with the definition of TLS (Figure 4G,H). These findings indicate that Cluster 6 is spatially and transcriptionally consistent with TLS.

**Figure 5:**
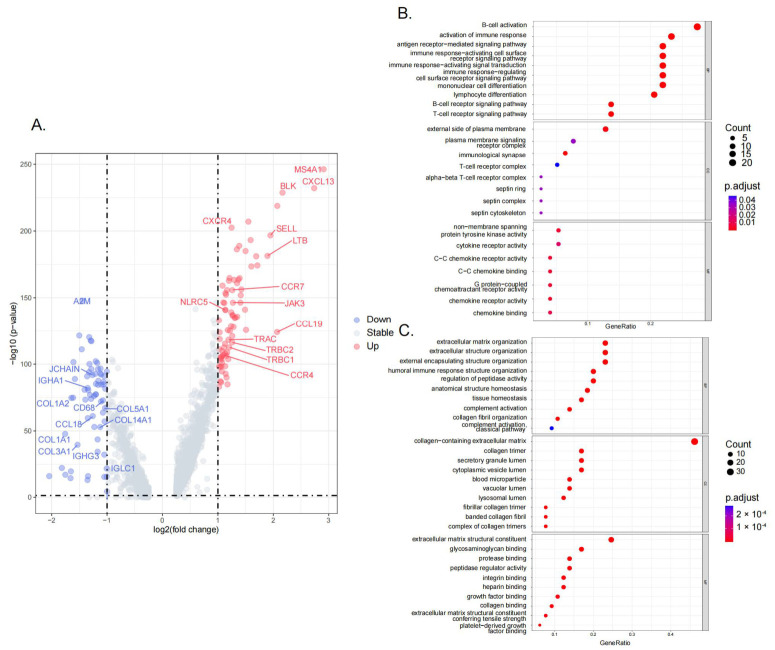

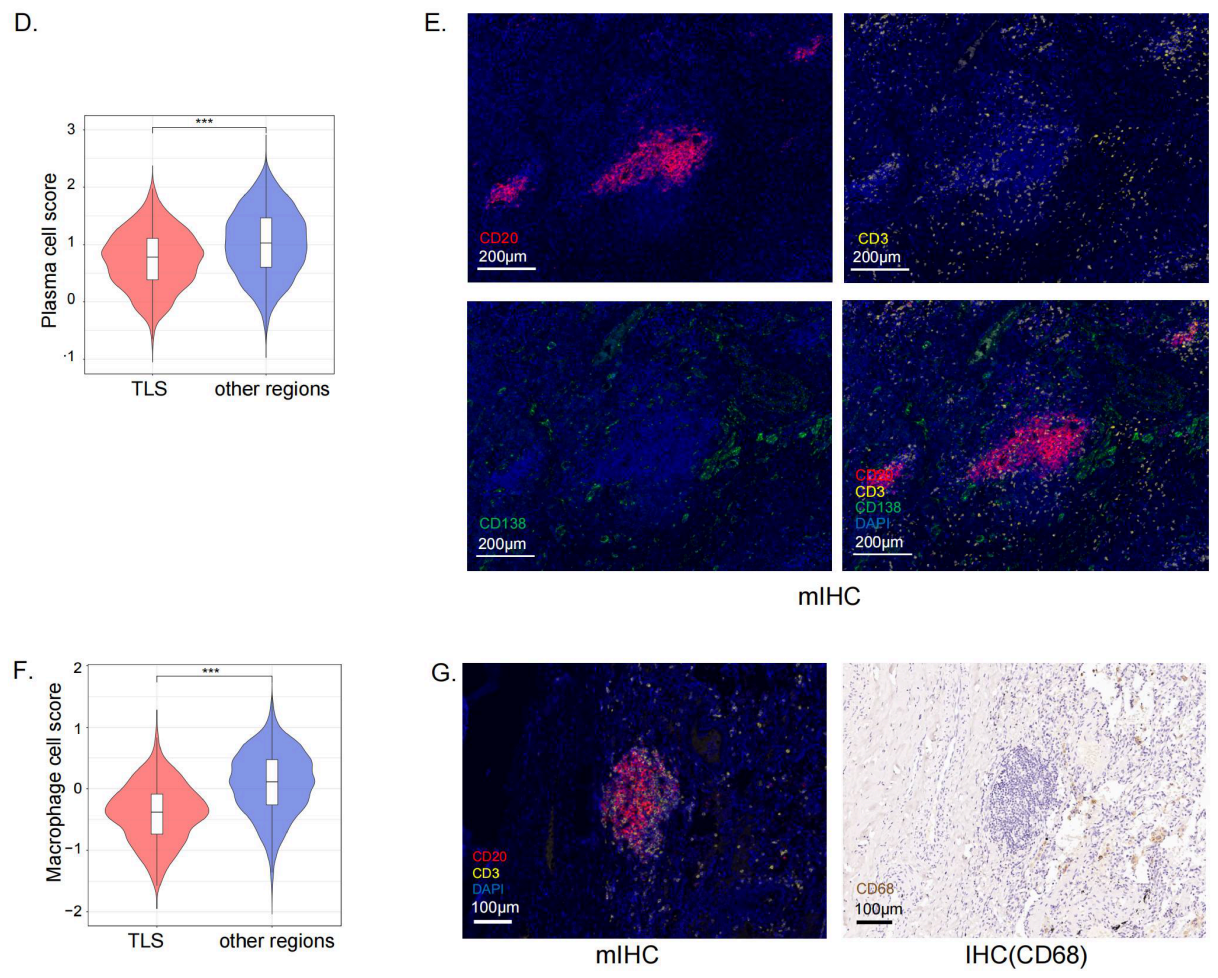
Activated B and T cells localized inside TLS while plasma cells and macrophages reside outside in the LUAD patient who responded to neoadjuvant therapy, in line with the observation in the LUSC patient (Figure 3). (**A**) Volcano plot of differentially expressed genes between TLS and other regions. Red dots denote genes significantly upregulated in TLS (|log2(fold change)| ≥ 1, adjusted *p* < 0.05); blue dots denote genes significantly downregulated in TLS; gray dots denote non-significance. |log_2_FC| = 1 (vertical dashed line) and *p* = 0.05 (horizontal dashed line, on the −log_10_ scale). (**B**,**C**) Gene ontology (GO) enrichment analysis of genes (**B**) upregulated in TLS and (**C**) downregulated in TLS (adjusted *p* < 0.05). (**D**) Violin plot for plasma cell score comparison between TLS and other regions. In the box, the center line denotes the median, and the box bounds represent the interquartile range. Wilcoxon rank-sum test was used; ***: *p* < 0.001. (**E**) mIHC staining on an individual LUAD specimen responded to neoadjuvant therapy. CD20 (B cells, top left) in red, CD3 (T cells, top right) in yellow, CD138 (plasma cells, button left) in green and nuclei were stained with DAPI (blue). Scale bar: 100 μm. (**F**) Violin plot for macrophage score comparison between TLS and other regions. In the box, the center line denotes the median, and the box bounds represent the interquartile range. Wilcoxon rank-sum test was used; ***: *p* < 0.001. (**G**) mIHC staining (left) and CD68 immunohistochemistry staining (brown, right) on an individual LUAD specimen responded to neoadjuvant therapy. CD20 in red, CD3 in yellow and nuclei were stained with DAPI (blue). Scale bar: 200 μm.

We then marked Cluster 6 as TLS. We performed an analysis of differentially expressed genes inside and outside TLS (Figure 5A). We also see that B- and T-cell-specific genes like MS4A1, TRAC, and TRBC1,2 were highly expressed inside TLS. *COL1A1*, *COL1A2*, and *COL3A1* which form type I and type III collagens were also highly expressed outside TLS. In line with previous findings, immunoglobulin advanced in development similar to IGHA1 and IGHG3 which was highly expressed outside TLS.

GO enrichment analysis was then conducted on genes highly expressed inside TLS and those outside as before (Figure 5B). Consistent with earlier results, GO terms related to “B cell activation” were also enriched in BP. GO terms associated with “activation of immune response” were enriched in TLS, including those related to B-cell and T-cell activating cell surface receptor signaling pathways. These enrichments indicate that B and T cells are likely physiologically activated—rather than resting within TLS, and also suggest that those immune cells were activated within TLS via cell surface receptor signaling.

Genes highly expressed outside TLS show enrichment in extracellular matrix/structure organization, which means that outside the TLS are mainly tissue cells of the lung or fibroblasts (Figure 5C). We can also see enrichment in “humoral immune response structure organization”, which implies the distribution of plasma cells.

Therefore, similar to the previous comparison in LUAD, we compared the plasma cell score inside TLS and that outside it, and found that the score inside TLS was significantly lower than that outside TLS (Figure 5D). An additional specimen from a different LUAD patient who responded to neoadjuvant therapy supported this result (Figure 5E). Most CD138^+^ cells were predominantly localized outside of B- and T-cell aggregates, consistent with the spatial transcriptome analysis as well as the result in LUSC.

Also, the macrophages score inside the TLS was significantly lower than that outside the TLS (Figure 5F), which means that macrophages mainly localized outside TLS. Additional LUAD specimen IHC staining with CD68 supports this conclusion independently (Figure 5G).

In conclusion, activated B and T cells localize within TLS in LUAD, while plasma cells and macrophages reside outside.

### 3.5. Antigen-Presenting Machinery (APM)-Related Genes Were Highly Expressed in TLS Accompanied by a High Expression of Upstream and Downstream Genes of MHC Class I, Within TLS in the Tumor Bed of NSCLC Patients

Human leukocyte antigen (HLA), also known as major histocompatibility complex (MHC), were a group of genes responsible for antigen presenting [27]. The MHC class I genes consist of HLA-A/B/C 3 genes. Previous work from our lab has demonstrated that HLA-A^+^ TLS are present in Esophageal squamous cell carcinoma (ESCC) tissues. Tumors from ESCC patients who respond to immune checkpoint blockade (ICB) therapy elevated the expression of an antigen-presenting machinery (APM), especially in TLS regions [20]. Here, we also test whether the APM was activated and enriched in TLS regions.

APM signature contains the following genes: *HLA-A*, *HLA-B*, *HLA-C*, *B2M*, *TAP1*, *TAP2*, and *TAPBP*. Several probes were not included in the sequencing panel (*HLA-A*, *HLA-B*, and *HLA-C*). This is probably the reason why we failed to enrich APM in the GO analysis. However, genes that belong to cell surface receptor signaling pathway were enriched, which still hints that the pathways were activated (Figure 3B and Figure 5B). Therefore, we used the remaining four genes to calculate their overall distribution in the spatial transcriptome sequencing region. For ease of description, it will be referred to as “APM-related score” in the following text.

**Figure 6:**
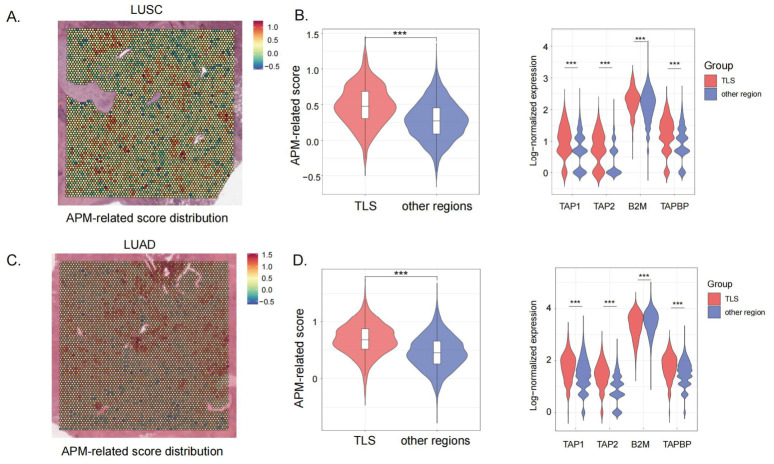

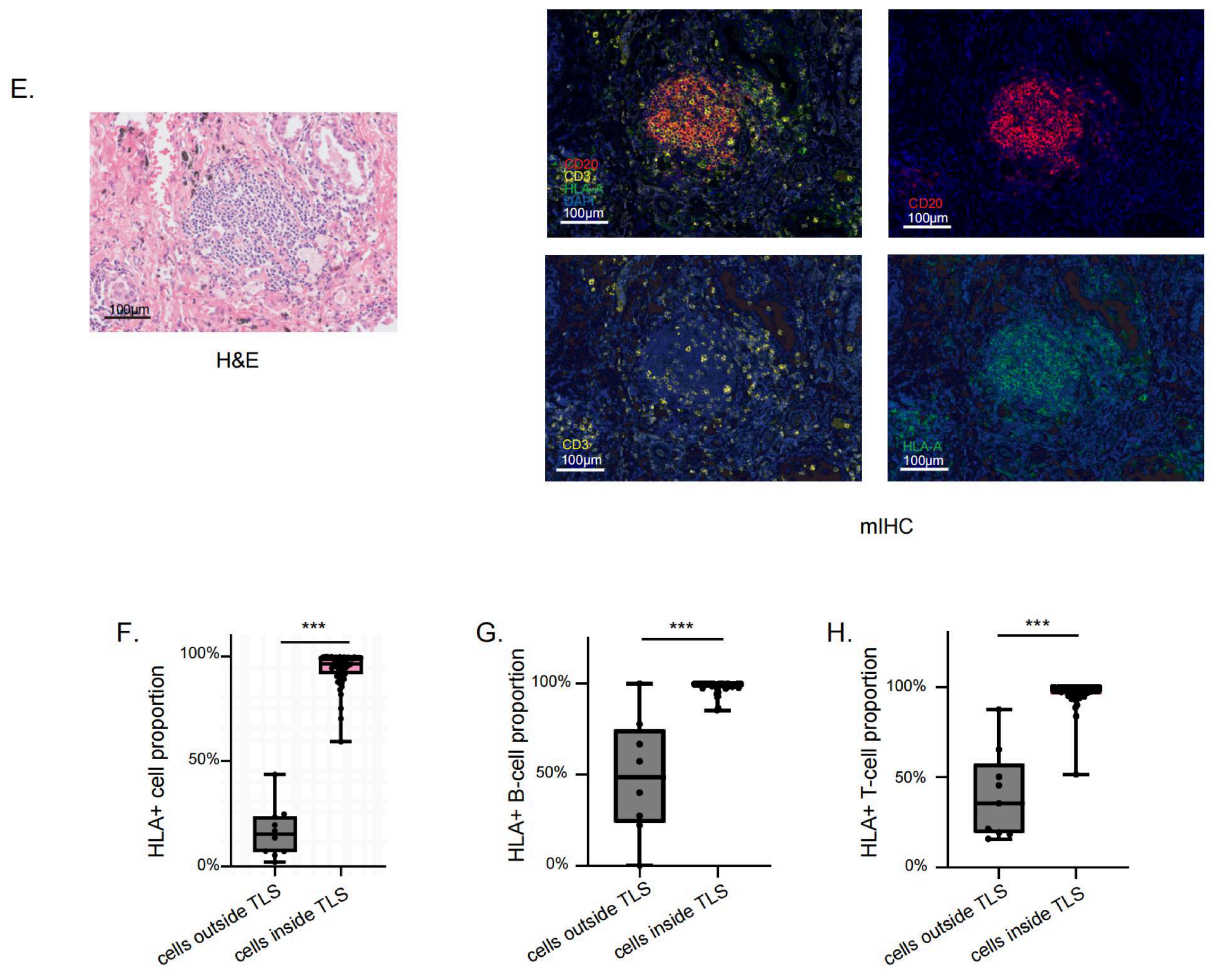
APM-related genes were highly expressed inside the TLS region than that outside the TLS region. (**A**–**D**) APM-related score distribution (including TAP1, TAP2, B2M, TAPBP). (**A**) Score overlaid on the LUSC H&E-stained section. (**B**) Violin plot for APM-related score (gene expression level) comparison between TLS and other regions in LUSC. Left: APM-related score comparison. Right: Genes included in APM-related score (TAP1, TAP2, B2M, TAPBP). (**C**) Score overlaid on the LUAD H&E-stained section. (**D**) Violin plot for APM-related score (gene expression level) comparison between TLS and other regions in LUAD. Left: APM-related score comparison. Right: Genes included in the APM-related score (TAP1, TAP2, B2M, TAPBP). The center line in the box denotes the median, and the box bounds represent the interquartile range. Wilcoxon rank-sum test was used; ***: *p* < 0.001. (**E**–**H**) Comparison of HLA-A expression conditions inside and outside the TLS. (**E**) Left: H&E staining of a TLS. Right: mIHC staining on a consecutive section shows the exact location of the TLS. CD20 (B cells) in red, CD3 (T cells) in yellow, HLA-A in green, and nuclei were counterstained with DAPI (blue). Scale bar: 100 μm. (**F**) Comparison of HLA-A positivity rate in all cells inside versus outside the TLS. (**G**) Comparison of HLA-A positivity rate in B cells inside versus outside the TLS. (**H**) Comparison of HLA-A positivity rate in T cells inside versus outside the TLS. For TLS-outside, 10 patients (five responders, five non-responders) were included; for each patient, three random fields were evaluated and the positivity rate was averaged. For TLS-inside, 66 patients with detectable TLS were analyzed. Each point represents one patient. Statistical significance was assessed using the Mann–Whitney test; ***: *p* < 0.001.

The spatial scatter plot showed that, in the LUSC specimen, the APM-related score distribution is close to the TLS score distribution (Figure 2D and Figure 6A). Furthermore, this score in TLS was significantly higher than outside TLS (Figure 6B left). A more detailed observation of the four genes showed that they had significantly higher expression levels within TLS than outside TLS (Figure 6B right).

The similar situation was observed in the LUAD sample. The spatial scatter plot also showed a spatial distribution pattern (Figure 4D and Figure 6C). The APM-related score was significantly higher in TLS (Figure 6D left). The four genes had significantly higher expression levels in TLS than outside TLS (Figure 6D right). Based on the above results, we can reasonably infer that the APM within the TLS has been activated, and the MHC I class molecules are likely to be in a highly expressed state.

To verify this conclusion, we perform mIHC staining of CD20, CD3, and HLA-A, together with DAPI on more samples from NSCLC patients who received neoadjuvant therapy and showed a response (Figure 6E).

By comparing the staining results of cells both inside and outside the TLS, it was found that the proportion of HLA-A^+^ cells inside the TLS was close to 100%, and this positive proportion was significantly higher than that of the cells outside the TLS (Figure 6F). The result was true for B and T cells: the proportion of HLA-A^+^ B cells and T cells within TLS is close to 100%, and it is significantly higher than that in B and T cells outside TLS (Figure 6G,H).

We further utilized spatial transcriptomic data to search for more evidence of APM activation, particularly for the activation of MHC class I genes. There is an SXY sequence upstream of the MHC class I genes, which is bound by multiple transcription factors that subsequently recruit NLRC5 (NLR family CARD domain-containing 5) [28]. Although NLRC5 itself lacks DNA-binding activity, it assembles with transcription factors into a coactivator complex known as the enhanceosome, which specifically drives MHC class I expression [29]. Our results showed that the expression level of NLRC5 in the TLS region of LUSC and LUAD is significantly higher than that outside the TLS (Figure S4A,B). This provides additional suggestive evidence that MHC class I genes were activated inside TLS, alongside the AMP-related genes.

*CD69* is the earliest surface antigen upregulated on T cells upon TCR engagement, which occurs when the TCR recognizes cognate peptide-MHC class I complexes presented by antigen-presenting cells [30]. We observed a significantly higher expression level of *CD69* in the TLS region of LUSC and LUAD than that outside the TLS (Figure S4C,D). This offers suggestive evidence that HLA-A is activated and functional. In conclusion, part of the APM-related genes (*B2M*, *TAP1*, *TAP2*, *TAPBP*) were highly expressed in TLS. A proportion of the HLA-A^+^ cells within the TLS region is higher than that outside the TLS. MHC class I genes within the TLS appear to be activated.

### 3.6. Heterogeneity Was Found Among TLS in NSCLC, Including Mature and Immature TLS Mature TLS Have a Larger Average Area Mainly by Accommodating More B Cells

To investigate whether there is heterogeneity among the TLS, we conducted additional IHC staining based on CD23 in consecutive sections from LUSC and LUAD patients.

**Figure 7:**
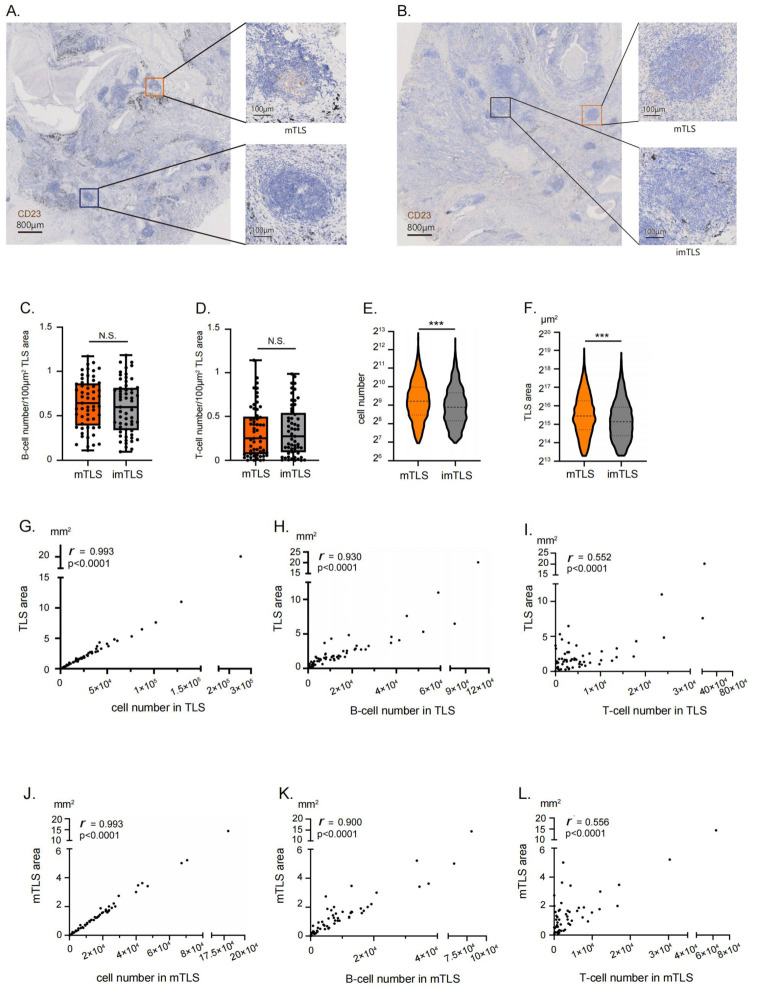
mTLS have larger areas and contain more B cells than imTLS. (**A**,**B**) CD23 immunohistochemistry (IHC) on serial sections from the same patients used for spatial transcriptomics. (**A**) LUSC specimen, (**B**) LUAD specimen. Scale bars: 800 μm (main image); 100 μm (inset). (**C**,**D**) Cell density comparison between mTLS and imTLS. (**C**) B-cell density (cells per 100 μm^2^ TLS area), (**D**) T-cell density (cells per 100 μm^2^ TLS area). Each point represents one patient. N.S., not significant (Mann–Whitney test; *p* > 0.05). (**E**,**F**) Comparison of (**E**) total cell number per TLS and (**F**) average TLS area (μm^2^) between mTLS and imTLS. Both total cell number and average area are extremely significantly larger in mTLS (Mann–Whitney test; ***: *p* < 0.001). In the violin plot, the center line denotes the median, and the upper/lower (dashed) lines denote the quartiles. A total of 1390 mTLS and 949 imTLS were included. (**G**–**I**) Correlation analysis between per-patient total TLS area and per-patient total cell counts for all TLS (including both mTLS and imTLS, *n* = 66). (**G**) Total cell number vs. area, (**H**) B-cell number vs. area, (**I**) T-cell number vs. area. Each point represents one patient. Spearman correlation coefficients (*r*) and *p* values are shown on each plot. (**J**–**L**) Correlation analysis between per-patient total cell counts and per-patient total mTLS area, restricted to mTLS only. (**J**) Total cell number vs. area, (**K**) B-cell number vs. area, (**L**) T-cell number vs. area. Each point represents one patient (*n* = 57 patients with at least one mTLS). Spearman correlation coefficients (*r*) and *p* values are shown on each plot.

The IHC staining results showed that there are mature TLS (mTLS) and immature TLS (imTLS) both in LUSC (Figure 7A) and LUAD (Figure 7B).

Then, mIHC and IHC staining on a cohort including 66 pathological samples of NSCLC patients were performed. mIHC was used to locate tertiary lymphoid structures (TLS) through dual staining with CD20 and CD3, whereas IHC staining for CD23 determined whether the TLS were mature. We distinguished mTLS and imTLS for each patient and normalized the total number of B and T cells within each TLS to the TLS area, which has been defined as the density of B cells and T cells within TLS.

The results showed that B cells had a slightly higher density in mTLS than imTLS with no significant differences (Figure 7C). There was no significant difference in the density of T cells between mTLS and imTLS (Figure 7D). The possible reason for these results is that TLS, as a dense tissue, cannot accommodate more cells on a unit area.

We then counted the number and area of cells in each mTLS and imTLS. The average number and area of cells in the mTLS were both greater than those in the imTLS group with an extremely significant difference (Figure 7E,F). This indicates that mature TLS develop a larger area by accommodating more cells.

To further clarify which type of cells made the primary contribution to the increase in mTLS area, we conducted separate correlation analyses between the TLS (mTLS) area and total cell number, B-cell number and T-cell number.

The Spearman rank correlation analysis revealed a strong positive monotonic association between TLS area and total cell count, with *r* = 0.993 (*p* < 0.0001) (Figure 7G). This result indicates that our results on cell counting and TLS area division are reliable. The B-cell number is also strongly positively correlated with the TLS area with a *p* value less than 0.0001 and an *r* value reaching 0.930 (Figure 7H). In contrast, the correlation between the TLS area and T-cell number was relatively weak (*r* = 0.552, *p* < 0.0001, Figure 7I). This result indicates that B cells instead of T cells are the primary determinant of changes in the TLS area.

In line with the result in TLS, the total cell number (Figure 7J) is positively correlated with the TLS area with a *p* value less than 0.0001 and an *r* value reaching 0.993. The correlation between B-cell counts and mTLS area was highly significant (*p* < 0.0001) with an *r* value reaching 0.900 (Figure 7K). At the same time, the correlation between the mTLS area and T-cell number was relatively weak (*r* = 0.556, *p* < 0.0001, Figure 7K). This result could reflect that B-cell accumulation directly promotes the expansion of TLS, and the T-cell number is not as tightly linked to the TLS area.

In conclusion, there is heterogeneity in the TLS within the tumor bed of NSCLC patients, including both mature and immature TLS. Mature TLS mainly expand their size by accommodating more B cells and, to a much lesser extent, a modest increase in T-cell numbers.

### 3.7. High Density and Area Proportion of TLS and Mature TLS in the Tumor Bed Are Associated with Favorable Response to Neoadjuvant Therapy, Possibly by Accommodating More B Cells Within the Tumor Bed

Finally, we wanted to know whether the density and area of these TLS were correlated with the efficacy of neoadjuvant therapy. For this purpose, the same cohort was used.

Here, the number of TLS on a certain area was defined as the density of TLS. When calculating the density and area ratio, TLS will be differentiated by its location.

**Figure 8:**
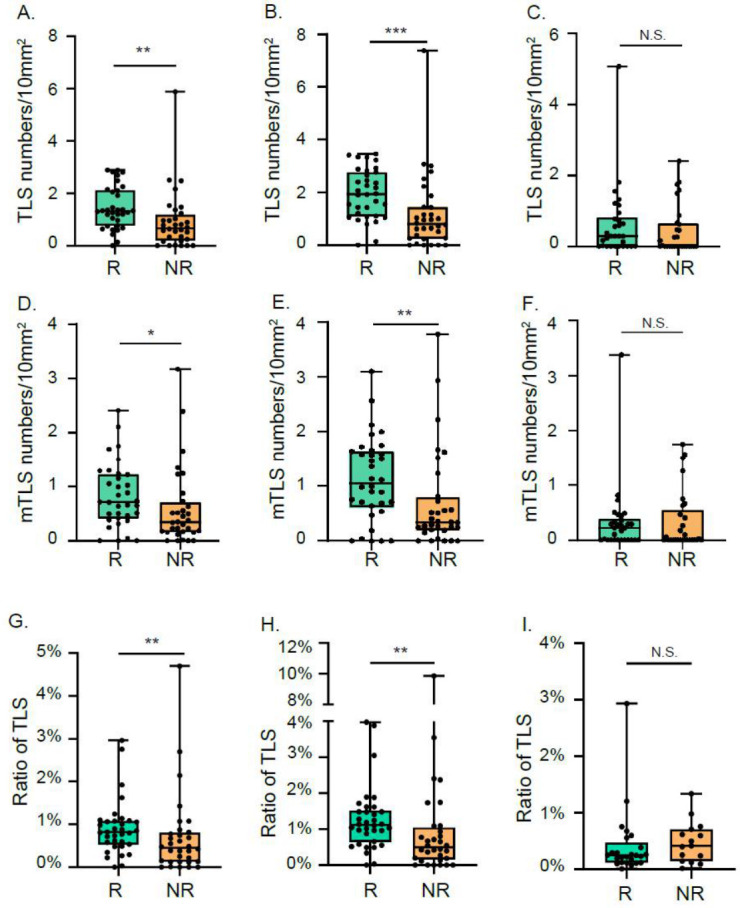

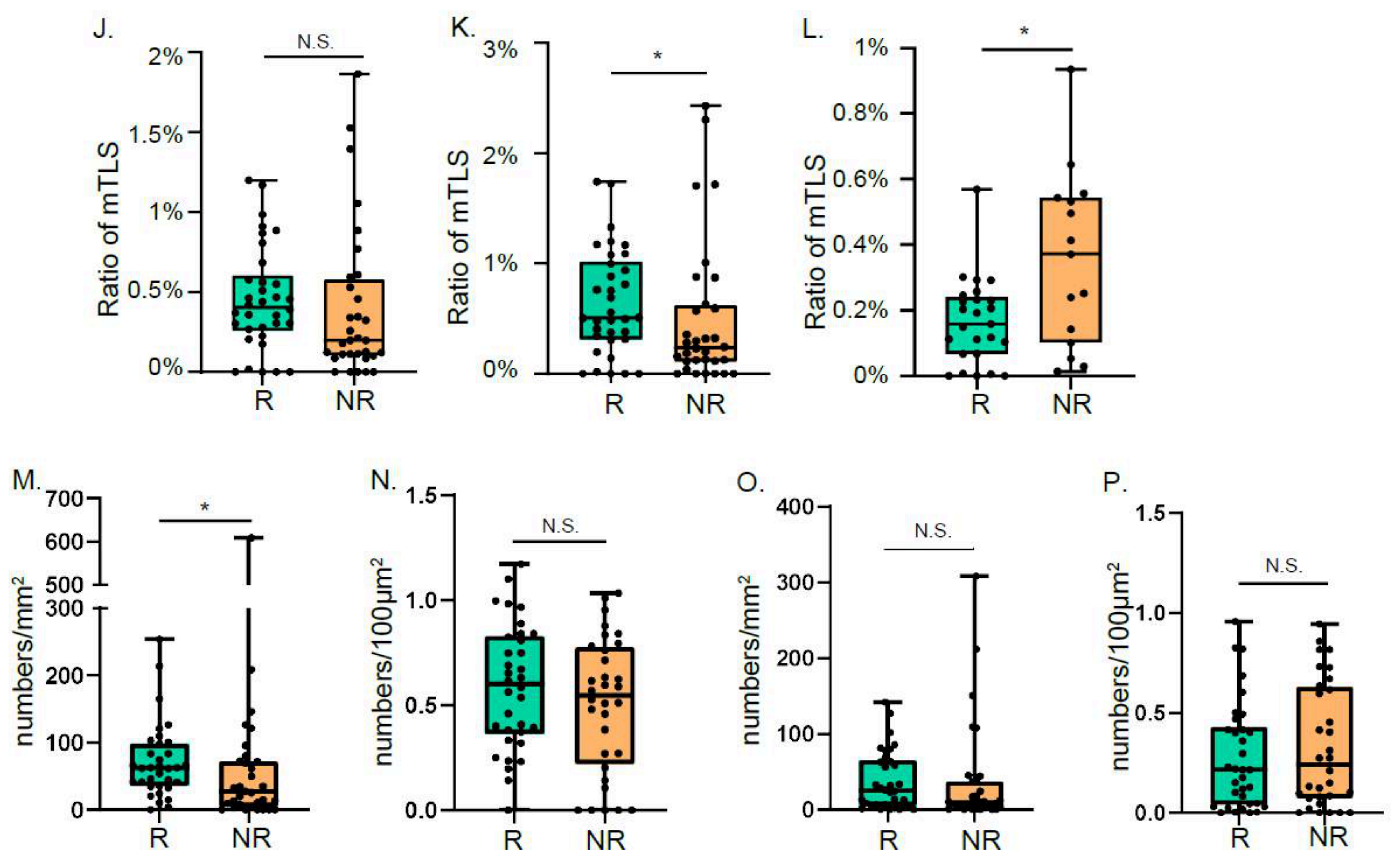
(m)TLS density and area proportion were associated with neoadjuvant therapy efficacy. (**A**–**C**) TLS density on the corresponding tissue. (**A**) TLS density on whole sample tissue. (**B**) TLS density on tumor bed. (**C**) TLS density on peritumoral tissue. (**D**–**F**) mTLS density on the corresponding tissue. (**D**) mTLS density on whole sample tissue. (**E**) mTLS density on tumor bed. (**F**) mTLS density on peritumoral tissue. (**G**–**I**) The area ratio of TLS on the corresponding tissue. (**G**) TLS area ratio on whole sample tissue. (**H**) TLS area ratio on tumor bed. (**I**) TLS area ratio on peritumoral tissue. (**J**–**L**) The area ratio of mTLS on the corresponding tissue. (**J**) mTLS area ratio on whole sample tissue. (**K**) mTLS area ratio on tumor bed. (**L**) mTLS area ratio on peritumoral tissue. (**M**–**P**) Density of cells residing within TLS in the corresponding tissue. (**M**) Density of TLS-resident B cells in the tumor region (B-cell number normalized to tumor area). (**N**) Density of TLS-resident B cells in the TLS region (B-cell number normalized to TLS area). (**O**) Density of TLS-resident T cells in the tumor region. (**P**) Density of TLS-resident T cells in the TLS region. Green: the responder group (*n* = 34); Orange: the non-responder group (*n* = 32). Each point represents one patient. Statistical significance was assessed using the Mann–Whitney test, N.S.: not significant; *: *p* < 0.05, **: *p* < 0.01, ***: *p* < 0.001.

The result showed that there were 1.45 ± 0.81 (mean ± SD, *n* = 34) TLS/10 mm^2^ in the responder group while 0.93 ± 1.14/10 mm^2^ (mean ± SD, *n* = 32) TLS in the non-responder group, which was significantly higher in the whole tissue area (Figure 8A). To enable a more detailed comparison, the sample tissue was divided into the tumor bed and the peritumoral tissue. TLS density in the tumor bed was 1.95 ± 0.95 TLS/10 mm^2^ in the responder group, significantly higher than that in the non-responder group (1.18 ± 1.15 TLS/10 mm^2^) (Figure 8B), whereas no significant difference was observed in the peritumoral tissue (0.62 ± 0.99 TLS/10 mm^2^, 0.44 ± 0.69 TLS/10 mm^2^) (Figure 8C).

Subsequently, the density of mTLS was introduced into the statistics. The result showed that there were 0.83 ± 0.59 mTLS/10 mm^2^ in the whole tissue area (Figure 8D) and 1.13 ± 0.77 mTLS/10 mm^2^ in the tumor bed (Figure 8E) in the responder group, which were significantly higher than that in the non-responder group separately (0.59 ± 0.72 mTLS/10 mm^2^, 0.70 ± 0.90 mTLS/10 mm^2^). However, in peritumoral tissue, the mTLS density had no significant differences (0.32 ± 0.62 mTLS/10 mm^2^ versus 0.33 ± 0.54 mTLS/10 mm^2^, Figure 8F).

The proportion of the TLS area was also analyzed. It was found that in the responder group, the proportion of TLS area normalized to tissue area was 0.91% ± 0.64% (mean ± SD, *n* = 34), significantly higher than that in the non-responder group (0.70% ± 0.95%, mean ± SD, *n* = 32) (Figure 8G). The proportion of TLS area normalized to tumor bed was 1.27% ± 0.90%, significantly higher than that in the non-responder group (1.01% ± 1.82%) as well (Figure 8H). However, the TLS area proportion to peritumoral tissue has no significant difference between the responder group and non-responder group (0.41% ± 0.59% versus 0.48% ± 0.37%, Figure 8I).

When the mTLS area ratio was introduced into the statistics, there was no significant difference observed between the responder and non-responder group normalized to the whole tumor tissue (0.46% ± 0.33% versus 0.40% ± 0.48%, Figure 8J). However, a significantly higher proportion of mTLS area normalized to tumor bed (0.64% ± 0.48%) in the responder group was observed (Figure 8K), while the area proportion of mTLS normalized to tumor bed in the non-responder group reached 0.50% ± 0.66%. Interestingly, in the non-responder group, a significantly higher proportion of mTLS area normalized to peritumor tissue was observed (0.17% ± 0.13% versus 0.35% ± 0.27%), indicating that the location of mTLS played an important role in the mechanism of neoadjuvant therapy response (Figure 8L).

Subsequently, we refined the data to the cellular level. The result showed that B cells belonging to TLS had a significantly higher density (71.32 ± 55.71 cells/mm^2^, mean ± SD, *n* = 34) in the responder group than the non-responder group (61.33 ± 111.95 cells/mm^2^, mean ± SD, *n* = 32) normalized to tumor bed area (Figure 8M). However, when these B cells are averaged only in TLS, there is no significant density difference between the responder group and non-responder group (0.59 ± 0.30 cells/100 μm^2^ versus 0.50 ± 0.32 cells/100 μm^2^, Figure 8N). No significant difference was observed between the responder and non-responder group in the average density of TLS-associated T cells, regardless of normalization to TLS area or tumor bed area (Figure 8O,P).

Based on this result, together with the conclusion of the previous section, it is likely to indicate that, as TLS develops, it becomes larger by mainly accommodating more B cells, gradually evolving into mTLS and thereby promoting the efficacy of neoadjuvant treatment for patients with NSCLC.

## 4. Discussion

In this study, spatial transcriptomic sequencing and analysis were performed on two patients with NSCLC (one case of LUSC and one case of LUAD) who exhibited a response to neoadjuvant therapy. We observed spatial heterogeneity of immune cells within the tumor bed. Activated B and T lymphocytes aggregated to form tertiary lymphoid structures, whereas macrophages and plasma cells were distributed outside the TLS.

A certain group of macrophages have been reported to secrete CXCL13 [31]. CXCL13 chemotaxis is essential in the formation of TLS, which can guide T and B lymphocytes to aggregate and form TLS. As TLS develops, B cells will differentiate into plasma cells, migrate out of TLS, and secrete antibodies, exerting anti-tumor effects [23]. Macrophages can also exert anti-tumor effects through antibody-dependent cellular cytotoxicity (ADCC). Antibodies secreted by plasma cells in turn recruit macrophages and kill tumors through ADCC action [23]. In this situation, this group of macrophages and plasma cells both have a positive impact on prognosis in neoadjuvant treatment.

Regarding plasma cells, Meylan et al. (2022) traced the full B-cell-to-plasma-cell differentiation program in renal cell carcinoma (RCC) [23]. Within TLS, B cells undergo clonal expansion, selection, and maturation into antibody-secreting plasma cells, which then migrate along fibroblast-derived tracks to the tumor, where their secreted IgG antibodies bind tumor cells and induce apoptosis. Although our data cannot trace Ig sequences to define migration routes, the observed heterogeneity in cell distribution is in line with the pattern in the literature.

A recent study in lung cancer [32] showed that LUAD primarily engages T cells and macrophages against tumors, whereas LUSC relies on B cells and plasma cells. This implies distinct anti-tumor mechanisms between the two non-small cell lung cancer subtypes, which is why we did not combine them for analysis.

Subtypes of tumor-associated macrophages play completely different roles in tumor microenvironment. M1 macrophages have a proinflammatory phenotype and have an anti-tumor effect, whereas M2 macrophages promote tumor development [33]. Taking macrophage as an example, spatial transcriptomics, while powerful, does not achieve a single-cell resolution this time. Spot-level measurements average out heterogeneity within a spot. The molecular mechanism of cell–cell interactions cannot be analyzed in more detail due to the lack of resolution. Extracting precise intercellular physical distances were not allowed due to the same reason. With the advancement of technology, platforms like 10X Xenium, Vizgen MERSCOPE, and Nanostring CosMx are expected to overcome these problems [34]. Also, the relatively small sample size limits the generalizability of our findings.

Inside the TLS, antigen-presenting machinery (APM) signature-related genes were highly expressed than that outside. Staining analysis of one cohort verified that HLA-A gene expression in cells within TLS was significantly higher than that outside TLS, with nearly 100% of cells being HLA-A^+^. Both *NLRC5* (an upstream activator of MHC class I) and *CD69* (an early T-cell activation marker triggered by TCR–peptide–MHC class I engagement) showed a significantly higher expression within TLS than outside.

NLRC5, together with the RFX complex can form the enhanceosome, binding to upstream of MHC class I genes, specifically activating the expression of HLA-A/B/C [35]. Based on the data from the Cancer Genome Atlas (TCGA), it was observed that the expression level of *NLRC5* was highly correlated with MHC class I in all 21 tumor types, as well as cytotoxic T-cell markers [36]. The elevated NLRC5 expression in TLS in our result is consistent with the higher HLA-A staining positivity observed in these regions, suggesting a local enhancement of MHC class I antigen-presenting machinery.

As we all know, activation of T cells requires the support of two signals. One of them is the formation of the immune synapse between TCR and peptide-MHC class I molecules [37]. Indeed, we observed that the T-cell receptor signaling pathway was enriched in both of the two samples (Figure 3D and Figure 5D). CD69, a primary marker for T-cell activation, has a higher expression level in TLS in our results. Beyond indicating the potential upregulation of MHC class I molecules, this elevated expression further indicates its functional role in the local immune microenvironment.

Our lab has previously shown that the number of HLA-A^+^ TLS is positively correlated with the density of re-activated CD8^+^ T cells in esophageal squamous cell carcinoma (ESCC) tissues [20]. In ESCC patients who respond to immune checkpoint blockade (ICB), tumors exhibit an upregulated expression of APM components, particularly within TLS regions [20]. TLS with high expression of HLA-A can promote the reactivation of CD8^+^ T cells, thereby enhancing the efficacy of immunotherapy. This might be a potential molecular mechanism by which TLS contributes to the effectiveness of neoadjuvant treatment responses.

In addition to differences in cell distribution, heterogeneity was observed among TLS. Using CD23 staining to classify TLS as mature or immature, we have found that mTLS occupy a larger area than imTLS. This size difference is associated with a marked increase in B-cell numbers and, to a much lesser extent, a modest increase in T-cell numbers.

Analysis of the B-cell repertoire revealed clonal diversification, selection, and expansion within TLS, along with the presence of fully mature clonotypes at a distance [23]. Therefore, the increasing of B-cell number in mTLS may be due to the clone expansion. However, the short-read sequencing of the NovaSeq platform and the relatively high RNA degradation rate of this FFPE sample prevented us from clearly tracking the proliferation and migration routes of the clones. We are unable to determine whether the additional B cells were generated through clonal expansion or chemotaxis.

Finally, multiplex immunohistochemistry (mIHC) staining on the cohort of samples demonstrated that (m)TLS density, area proportion, and number of B lymphocytes in the tumor bed, as well as the area proportion of mTLS in the adjacent tissues, can be used as indicators for evaluating the efficacy of neoadjuvant therapy. Interestingly, the proportion of mTLS in the peritumoral area was higher in non-responder patients, which is similar to findings in hepatocellular carcinoma (HCC) [13]. In patients with HCC, the presence of TLS in adjacent tumor tissue is considered to create a protective niche for tumor progenitor cells, ultimately contributing to long-term recurrence (≥2 years). Our result also indicates the different locations of TLS in tissues have different associations with neoadjuvant therapeutic effects.

However, our retrospective study only demonstrates a correlation, rather than a causal relationship, between TLS features and therapy response. Therefore, functional experiments are required to establish causality. Beyond that, all patients in this study received neoadjuvant therapy based on anti-PD-1 therapy. Therefore, whether our findings extend to other immune checkpoint inhibitors not represented in our cohort (e.g., anti-CTLA-4 antibodies) remains to be determined. The applicability to other treatment modalities also requires further investigation. Meanwhile, due to the retrospective nature of specimen collection, some clinical covariates (e.g., smoking, PD-L1, treatment details) were not consistently recorded; thus, our findings are descriptive and should be validated in prospective cohorts.

## 5. Conclusions

In conclusion, we conducted spatial transcriptome sequencing and found TLS in the NSCLC tumor bed. The two patients who responded to neoadjuvant therapy showed that activated B and T cells localized inside TLS and plasma cells/macrophages were mainly outside. APM-related genes were upregulated in TLS compared to other regions, along with elevated upstream and downstream genes of MHC class I. TLS have heterogeneity and include both mature and immature TLS. mTLS have a larger area by mainly containing more B cells. Higher TLS and mature TLS density, as well as area proportion, particularly B-cell enrichment within mature TLS, are associated with promising neoadjuvant therapy response in NSCLC.

Our results implicate a potential mechanism underlying the role of TLS in neoadjuvant therapy for NSCLC. High APM-related genes, together with NLRC5, stimulated the MHC class I, especially HLA-A. The APM expression in TLS may activate T cells and promotes tumor killing. Meanwhile, sustained antigenic stimulation drives the development of TLS into mTLS via a greater number of B cells and a slight contribution of T cells. B cells also underwent proliferation and activation in TLS. After that, the plasma cells could migrate into tissues and secrete antibodies, which may recruit macrophages to exert ADCC-mediated tumor destruction, thereby conferring a clinical benefit to neoadjuvant therapy recipients. This work points to several promising directions for subsequent investigation. While further functional validation is needed, our study offers a novel mechanistic framework linking TLS maturation to clinical response in NSCLC.

## Data Availability

The datasets generated and/or analyzed during the current study are available from the corresponding author on reasonable request.

## Supplementary Materials

The following supporting information can be downloaded at: https://www.mdpi.com/article/10.3390/cancers18132141/s1, Figures S1–S4: QC_and_Supplementary_results; Table S1: Summary Table of Patient Characteristics.

## Abbreviations

ADCC	Antibody-dependent cellular cytotoxicity
APM	Antigen-presenting machinery
ESCC	Esophageal squamous cell carcinoma
H&E	Hematoxylin-eosin staining
HLA	Human leukocyte antigen
ICB	Immune checkpoint blockade
IHC	Immunohistochemistry staining
LUAD	Lung adenocarcinoma
LUSC	Lung squamous cell carcinoma
mIHC	Multiplex immunohistochemistry staining
MHC	Major histocompatibility complex
NSCLC	Non-small cell lung cancer
RCC	Renal cell carcinoma
TLS	Tertiary lymphoid structures
m/imTLS	mature/immature TLS

## References

[1] Leiter A., Veluswamy R.R., Wisnivesky J.P. (2023). The Global Burden of Lung Cancer: Current Status and Future Trends. Nat. Rev. Clin. Oncol..

[2] Hendriks L.E.L., Remon J., Faivre-Finn C., Garassino M.C., Heymach J.V., Kerr K.M., Tan D.S.W., Veronesi G., Reck M. (2024). Non-Small-Cell Lung Cancer. Nat. Rev. Dis. Primers.

[3] Wang H., Pang J., Zhang S., Yu Q., Chen Y., Wang L., Sheng M., Dan J., Tang W. (2023). Single-Cell and Bulk RNA-Sequencing Analysis to Predict the Role and Clinical Value of CD36 in Lung Squamous Cell Carcinoma. Heliyon.

[4] Lu Y., Yang A., Quan C., Pan Y., Zhang H., Li Y., Gao C., Lu H., Wang X., Cao P. (2022). A Single-Cell Atlas of the Multicellular Ecosystem of Primary and Metastatic Hepatocellular Carcinoma. Nat. Commun..

[5] Mentucci F.M., Ferrara M.G., Ercole A., Rumie Vittar N.B., Lamberti M.J. (2025). Interplay between Cancer-Associated Fibroblasts and Dendritic Cells: Implications for Tumor Immunity. Front. Immunol..

[6] Dieu-Nosjean M.-C., Antoine M., Danel C., Heudes D., Wislez M., Poulot V., Rabbe N., Laurans L., Tartour E., de Chaisemartin L. (2008). Long-Term Survival for Patients with Non-Small-Cell Lung Cancer with Intratumoral Lymphoid Structures. J. Clin. Oncol..

[7] Zhao L., Jin S., Wang S., Zhang Z., Wang X., Chen Z., Wang X., Huang S., Zhang D., Wu H. (2024). Tertiary Lymphoid Structures in Diseases: Immune Mechanisms and Therapeutic Advances. Signal Transduct. Target. Ther..

[8] Cabrita R., Lauss M., Sanna A., Donia M., Skaarup Larsen M., Mitra S., Johansson I., Phung B., Harbst K., Vallon-Christersson J. (2020). Tertiary Lymphoid Structures Improve Immunotherapy and Survival in Melanoma. Nature.

[9] Helmink B.A., Reddy S.M., Gao J., Zhang S., Basar R., Thakur R., Yizhak K., Sade-Feldman M., Blando J., Han G. (2020). B Cells and Tertiary Lymphoid Structures Promote Immunotherapy Response. Nature.

[10] Petitprez F., de Reyniès A., Keung E.Z., Chen T.W.-W., Sun C.-M., Calderaro J., Jeng Y.-M., Hsiao L.-P., Lacroix L., Bougoüin A. (2020). B Cells Are Associated with Survival and Immunotherapy Response in Sarcoma. Nature.

[11] Gao J., Navai N., Alhalabi O., Siefker-Radtke A., Campbell M.T., Tidwell R.S., Guo C.C., Kamat A.M., Matin S.F., Araujo J.C. (2020). Neoadjuvant PD-L1 plus CTLA-4 Blockade in Patients with Cisplatin-Ineligible Operable High-Risk Urothelial Carcinoma. Nat. Med..

[12] Siliņa K., Soltermann A., Attar F.M., Casanova R., Uckeley Z.M., Thut H., Wandres M., Isajevs S., Cheng P., Curioni-Fontecedro A. (2018). Germinal Centers Determine the Prognostic Relevance of Tertiary Lymphoid Structures and Are Impaired by Corticosteroids in Lung Squamous Cell Carcinoma. Cancer Res..

[13] Sautès-Fridman C., Petitprez F., Calderaro J., Fridman W.H. (2019). Tertiary Lymphoid Structures in the Era of Cancer Immunotherapy. Nat. Rev. Cancer.

[14] Hellmann M.D., Chaft J.E., William W.N., Rusch V., Pisters K.M.W., Kalhor N., Pataer A., Travis W.D., Swisher S.G., Kris M.G. (2014). Pathological Response after Neoadjuvant Chemotherapy in Resectable Non-Small-Cell Lung Cancers: Proposal for the Use of Major Pathological Response as a Surrogate Endpoint. Lancet Oncol..

[15] Qi J., Sun H., Zhang Y., Wang Z., Xun Z., Li Z., Ding X., Bao R., Hong L., Jia W. (2022). Single-Cell and Spatial Analysis Reveal Interaction of FAP+ Fibroblasts and SPP1+ Macrophages in Colorectal Cancer. Nat. Commun..

[16] Vanhersecke L., Brunet M., Guégan J.-P., Rey C., Bougouin A., Cousin S., Moulec S.L., Besse B., Loriot Y., Larroquette M. (2021). Mature Tertiary Lymphoid Structures Predict Immune Checkpoint Inhibitor Efficacy in Solid Tumors Independently of PD-L1 Expression. Nat. Cancer.

[17] Barmpoutis P., Di Capite M., Kayhanian H., Waddingham W., Alexander D.C., Jansen M., Kwong F.N.K. (2021). Tertiary Lymphoid Structures (TLS) Identification and Density Assessment on H&E-Stained Digital Slides of Lung Cancer. PLoS ONE.

[18] Huang Z.-Y., Shao M.-M., Zhang J.-C., Yi F.-S., Du J., Zhou Q., Wu F.-Y., Li S., Li W., Huang X.-Z. (2021). Single-Cell Analysis of Diverse Immune Phenotypes in Malignant Pleural Effusion. Nat. Commun..

[19] Patil N.S., Nabet B.Y., Müller S., Koeppen H., Zou W., Giltnane J., Au-Yeung A., Srivats S., Cheng J.H., Takahashi C. (2022). Intratumoral Plasma Cells Predict Outcomes to PD-L1 Blockade in Non-Small Cell Lung Cancer. Cancer Cell.

[20] Zhang D., Jiang D., Jiang L., Ma J., Wang X., Xu X., Chen Z., Jiang M., Ye W., Wang J. (2024). HLA-A+ Tertiary Lymphoid Structures with Reactivated Tumor Infiltrating Lymphocytes Are Associated with a Positive Immunotherapy Response in Esophageal Squamous Cell Carcinoma. Br. J. Cancer.

[21] Rodriguez A.B., Peske J.D., Woods A.N., Leick K.M., Mauldin I.S., Meneveau M.O., Young S.J., Lindsay R.S., Melssen M.M., Cyranowski S. (2021). Immune Mechanisms Orchestrate Tertiary Lymphoid Structures in Tumors via Cancer-Associated Fibroblasts. Cell Rep..

[22] Liu L., Stephens B., Bergman M., May A., Chiang T. (2021). Role of Collagen in Airway Mechanics. Bioengineering.

[23] Meylan M., Petitprez F., Becht E., Bougoüin A., Pupier G., Calvez A., Giglioli I., Verkarre V., Lacroix G., Verneau J. (2022). Tertiary Lymphoid Structures Generate and Propagate Anti-Tumor Antibody-Producing Plasma Cells in Renal Cell Cancer. Immunity.

[24] Kumar S., Wuerffel R., Achour I., Lajoie B., Sen R., Dekker J., Feeney A.J., Kenter A.L. (2013). Flexible Ordering of Antibody Class Switch and V(D)J Joining during B-Cell Ontogeny. Genes. Dev..

[25] Germain C., Gnjatic S., Tamzalit F., Knockaert S., Remark R., Goc J., Lepelley A., Becht E., Katsahian S., Bizouard G. (2014). Presence of B Cells in Tertiary Lymphoid Structures Is Associated with a Protective Immunity in Patients with Lung Cancer. Am. J. Respir. Crit. Care Med..

[26] Colvin K.L., Cripe P.J., Ivy D.D., Stenmark K.R., Yeager M.E. (2013). Bronchus-Associated Lymphoid Tissue in Pulmonary Hypertension Produces Pathologic Autoantibodies. Am. J. Respir. Crit. Care Med..

[27] Klein J., Sato A. (2000). The HLA System. First of Two Parts. N. Engl. J. Med..

[28] Jongsma M.L.M., Neefjes J., Spaapen R.M. (2021). Playing Hide and Seek: Tumor Cells in Control of MHC Class I Antigen Presentation. Mol. Immunol..

[29] Dersh D., Phelan J.D., Gumina M.E., Wang B., Arbuckle J.H., Holly J., Kishton R.J., Markowitz T.E., Seedhom M.O., Fridlyand N. (2021). Genome-Wide Screens Identify Lineage- and Tumor-Specific Genes Modulating MHC-I- and MHC-II-Restricted Immunosurveillance of Human Lymphomas. Immunity.

[30] del Carmen Castellanos M., López-Giral S., López-Cabrera M., de Landázuri M.O. (2002). Multiple Cis-Acting Elements Regulate the Expression of the Early T Cell Activation Antigen CD69. Eur. J. Immunol..

[31] Carlsen H.S., Baekkevold E.S., Morton H.C., Haraldsen G., Brandtzaeg P. (2004). Monocyte-like and Mature Macrophages Produce CXCL13 (B Cell-Attracting Chemokine 1) in Inflammatory Lesions with Lymphoid Neogenesis. Blood.

[32] Enfield K.S.S., Colliver E., Lee C., Magness A., Moore D.A., Sivakumar M., Grigoriadis K., Pich O., Karasaki T., Hobson P.S. (2024). Spatial Architecture of Myeloid and T Cells Orchestrates Immune Evasion and Clinical Outcome in Lung Cancer. Cancer Discov..

[33] Toledo B., Zhu Chen L., Paniagua-Sancho M., Marchal J.A., Perán M., Giovannetti E. (2024). Deciphering the Performance of Macrophages in Tumour Microenvironment: A Call for Precision Immunotherapy. J. Hematol. Oncol..

[34] Wang H., Huang R., Nelson J., Gao C., Tran M., Yeaton A., Krishna S., Felt K., Pfaff K.L., Bowman T. (2025). Systematic Benchmarking of Imaging Spatial Transcriptomics Platforms in FFPE Tissues. Nat. Commun..

[35] Neerincx A., Rodriguez G.M., Steimle V., Kufer T.A. (2012). NLRC5 Controls Basal MHC Class I Gene Expression in an MHC Enhanceosome-Dependent Manner. J. Immunol..

[36] Yoshihama S., Roszik J., Downs I., Meissner T.B., Vijayan S., Chapuy B., Sidiq T., Shipp M.A., Lizee G.A., Kobayashi K.S. (2016). NLRC5/MHC Class I Transactivator Is a Target for Immune Evasion in Cancer. Proc. Natl. Acad. Sci. USA.

[37] Saveanu L., Zucchetti A.E., Evnouchidou I., Ardouin L., Hivroz C. (2019). Is There a Place and Role for Endocytic TCR Signaling?. Immunol. Rev..

