# Supplementary material for: Spatial Transcriptomics of Immune Cell Distribution in Non-Small Cell Lung Cancer Identifies Tertiary Lymphoid Structures and Its Density and Area Fraction Were Associated with Neoadjuvant Therapy Response
Source: Cancers (Basel). 2026 Jul 2;18(13):2141. doi: 10.3390/cancers18132141 (PMC13359995; doi:10.3390/cancers18132141)
Supplement: Supplementary file 1 [file cancers-18-02141-s001.zip › Supplementary Figure1~4_QC_and_Supplementary_results.pdf]

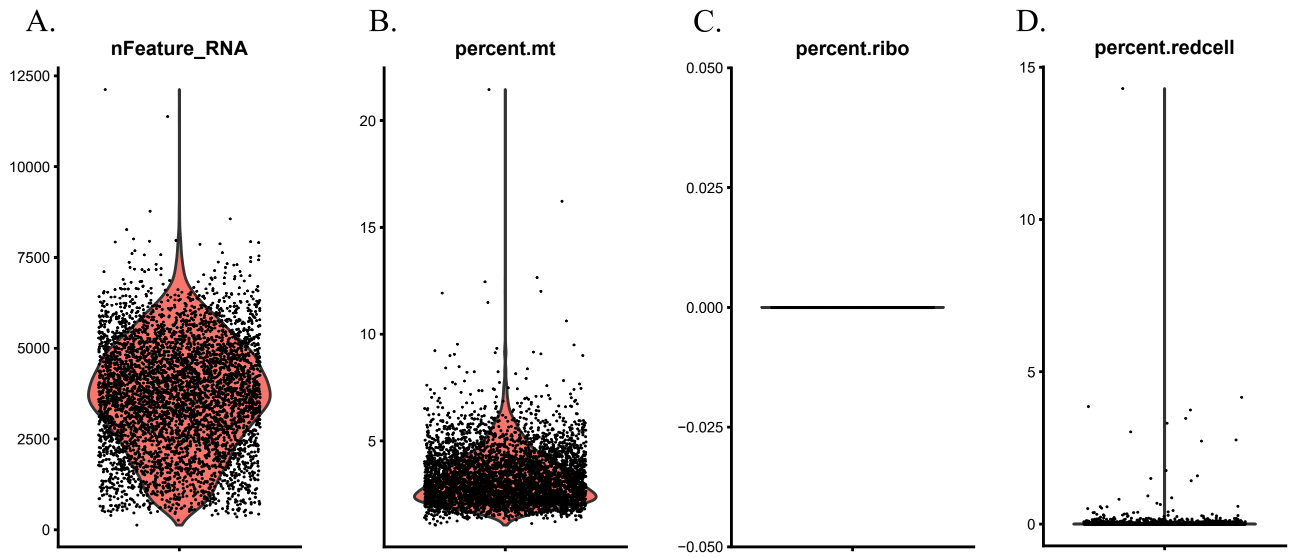

Supplementary Figure S1 . Quality control of FFPE samples from a LUSC patient after spatial transcriptome sequencing.
(A) nFeature number; (B) percentage of mitochondrial counts; (C) percentage of ribosom counts ;(D)percentage of redcell gene counts;

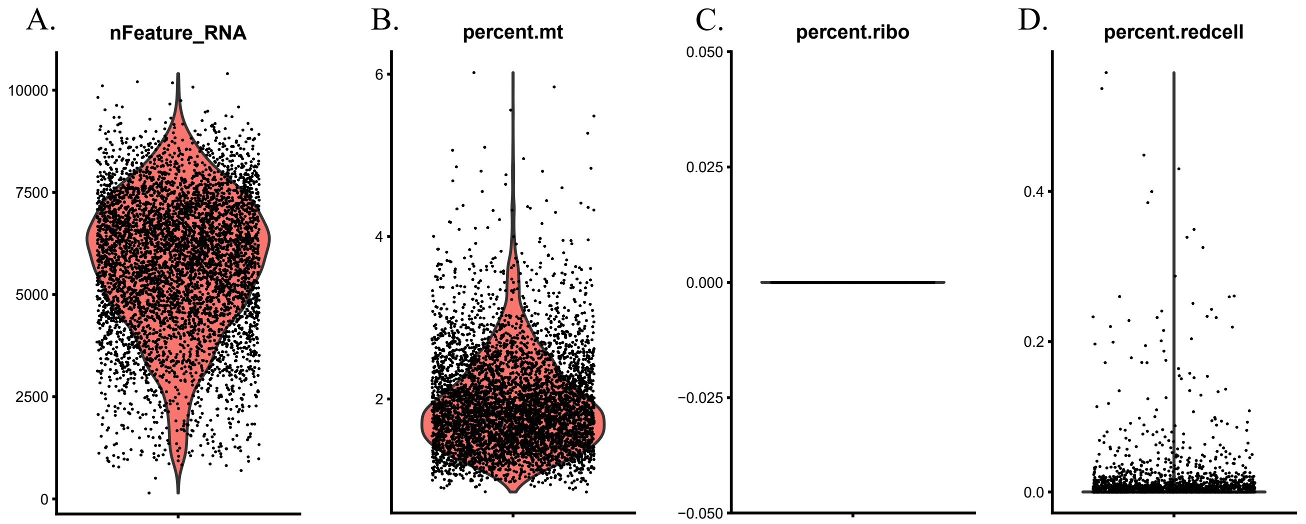

Supplementary Figure S2 . Quality control of FFPE samples from a LUAD patient after spatial transcriptome sequencing.
(A) nFeature number; (B) percentage of mitochondrial counts; (C) percentage of ribosom counts ;(D)percentage of redcell gene counts;

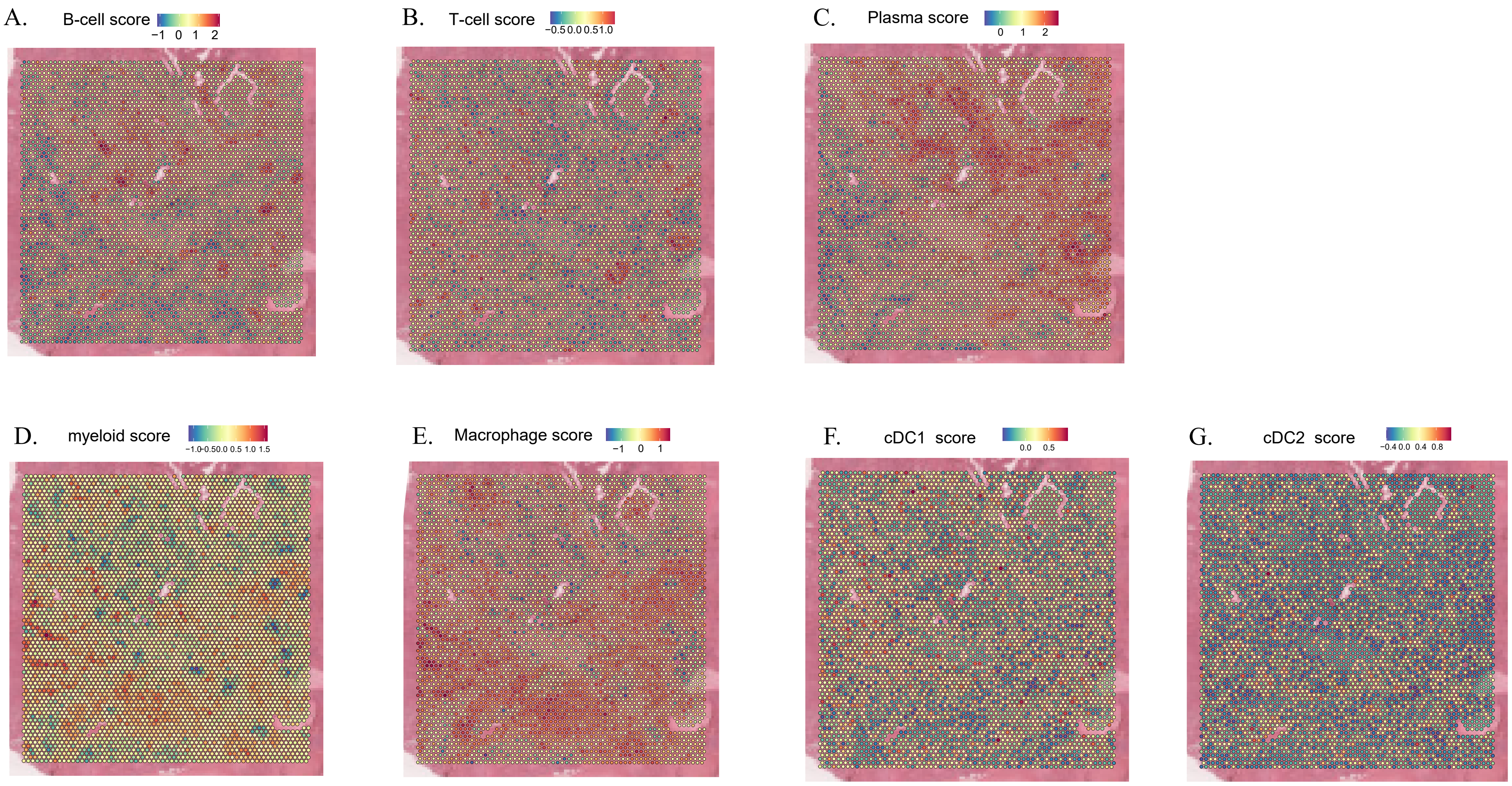

Supplementary Figure S3 . Spatial distribution of different immune cell score overlaid on the H&E-stained tissue section for a single LUAD patient responding to neoadjuvant therapy. (A) Spatial distribution of B-cell scores overlaid on the H&E-stained tissue section. (B) T-cell scores, (C) Plasma-cell scores, (D) Myeloid scores, (E) Macrophage scores, (F) conventional dendritic cell type 1 (cDC1) scores, (G) cDC2 scores.

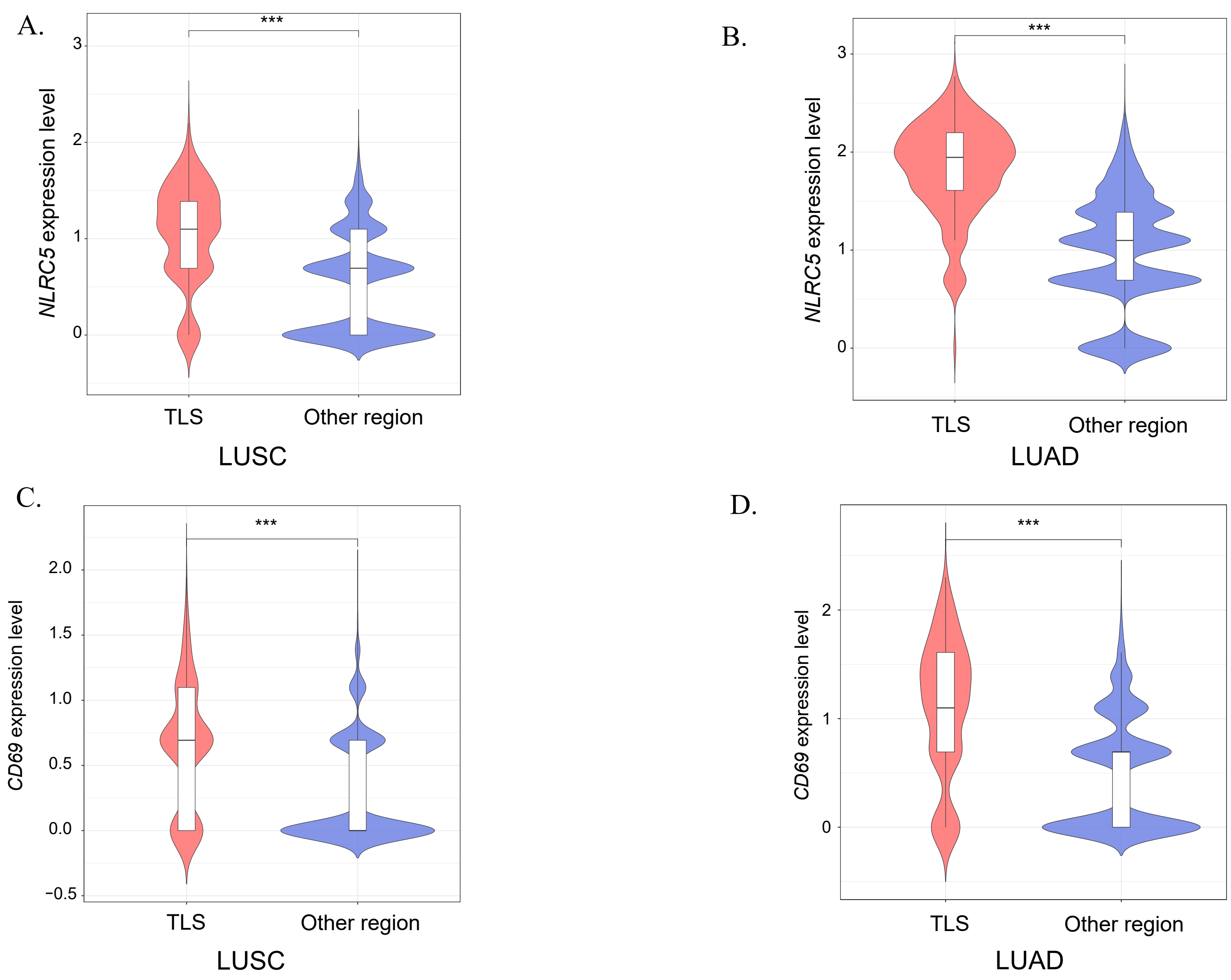

Supplementary Figure S4 . Suggestive evidence of elevated HLA expression in TLS. (A) Violin plot comparing NLRC5 expression levels inside and outside TLS in LUSC and (B) LUAD.(C) Violin plot comparing CD69 expression levels inside and outside TLS in LUSC and (D) LUAD. The center line in the box denotes the median, and the box bounds represent the interquartile range. Wilcoxon rank-sum test was used, ***: p＜0.001.
